# Exploring the link between eating disorders and persistent genital arousal disorder/genito-pelvic dysesthesia: first description and a systematic review of the literature

**DOI:** 10.1186/s40337-022-00687-7

**Published:** 2022-11-10

**Authors:** Hartmut Imgart, Annika Zanko, Sandra Lorek, Patti-Sue Schlichterle, Michael Zeiler

**Affiliations:** 1 Competence Center for Eating Disorders, Parkland Clinic, Im Kreuzfeld 6, 34537 Bad Wildungen, Germany; 2 Eating Disorder Unit, Department for Child and Adolescent Psychiatry, Medical University of Vienna, Waehringer Guertel 18-20, 1090 Vienna, Austria

**Keywords:** Persistent genital arousal disorder, Genito-pelvic dysesthesia, Eating disorders, Anorexia nervosa, Systematic review, Sexual dysfunction

## Abstract

**Background:**

Persistent Genital Arousal Disorder/Genito-Pelvic Dysesthesia (PGAD/GPD) characterized by recurrent physiological genital without corresponding psychological arousal is a poorly understood and researched condition. Based on the first two case descriptions of eating disorders directly linked to PGAD/GPD the aim of this paper was to systematically review the literature on possible associations between eating disorders and PGAD/GPD.

**Method:**

A systematic literature search on eating disorders and PGAD/GPD was conducted in PubMed, PsycINFO, and Scopus, complemented by Google Scholar. We included case reports, case series, cross-sectional studies and review articles published in peer-reviewed journals written in English or German-language.

**Results:**

The included original papers described a total of 2078 cases with PGAD/GPD symptomatology. Of these, 892 participants fulfilled all five PGAD/GPD core criteria. The aetiology of PGAD/GPD is unknown. Multifactorial genesis of PGAD/GPD is presumed including neurological, pharmacological, hormonal, vascular and psychological causes. A high degree of psychological comorbidity is reported. No study was found that drew a direct link between eating disorders and PGAD/GPD. Although PGAD/GPD symptoms also occur in adolescents, there are no findings in this regard. However, we found a gap in data collection: eating disorders as potential psychiatric comorbidities were systematically recorded in only a few studies.

**Conclusion:**

The existing literature have not yet considered a possible link between eating disorders and PGAD/GPD so far. According to the authors’ knowledge, this work is the first review to systematically explore the associations. We suspect underreporting of PGAD/GPD cases in eating disorders and particularly during adolescence. We argue that there are several common factors that appear to be important in the etiology, course, and treatment of both disorders (e.g. hormonal dysregulation or sensory sensitivity and avoidance), warranting future research on the possible comorbidity of these disorders.

**Supplementary Information:**

The online version contains supplementary material available at 10.1186/s40337-022-00687-7.

## Plain English summary

Persistent genital arousal disorder/genito-pelvic dysesthesia (PGAD/GPD) is characterized by recurrent physiological genital arousal without corresponding psychological arousal. PGAD/GPD is a poorly understood and researched disorder. It is often associated with significant psychological impairment and distress.

Eating disorders are severe psychological illnesses that predominantly affect the female sex. They have a high psychological comorbidity and are often associated with severe physical consequences.

We treated two female patients in whom there was a direct association between PGAD/GPD and eating disorder. In our systematic review of the literature, we found no study that further investigated the co-occurrence between PGADPGAD/GPD and eating disorders. This paper is the first to describe and investigate a relationship between PGAD/GPD and eating disorders.

In previous literature, several factors have been hypothesized for the development of PGAD/GPD but also eating disorders. We argue that there are several common factors that appear to be important in the etiology, course, and treatment of both disorders (e.g. hormonal dysregulation or sensory sensitivity and avoidance), warranting future research on the possible comorbidity of these disorders.

**Supplementary Information:**

The online version contains supplementary material available at 10.1186/s40337-022-00687-7.

## Introduction

In 2001, Leiblum and Nathan used the term “persistent sexual arousal syndrome” (PSAS) to first-time describe a condition with symptoms of unremitting genital arousal that are experienced in the absence of sexual desire without obvious hormonal, vascular, neurological or psychological causes [1]. The term was revised to “persistent genital arousal disorder” (PGAD) by Leiblum in 2006 in order to emphasize that it is rather a condition of genital than of sexual arousal [2]. PGAD is characterized by recurrent physiological genital arousal without corresponding psychological arousal [3, 4]. Genital arousal is predominantly experienced as involuntary and often results in significant psychological distress [4]. PGAD predominantly has been considered a sexual dysfunction [5].

For diagnosis, the original diagnostic criteria of Leiblum and Nathan [1] are mostly used. According to these authors, the following five criteria must be met for a diagnosis of PGAD to be made:

1. physiological responses characteristic of sexual arousal (genital and breast vasocongestion and sensitivity) that persist for an extended period of time (hours to days) and do not subsist completely on their own,
2. signs of physiological arousal that do not resolve with ordinary orgasmic experience and may require multiple orgasms over hours and days to remit,
3. physiological signs of arousal that are usually experienced as unrelated to any subjective sense of sexual excitement or desire,
4. persistent sexual arousal that may be triggered not only by a sexual activity, but also by seemingly non-sexual stimuli or by no apparent stimulus at all, and.
5. physiological signs of persistent arousal that are experienced as unbidden, intrusive, and unwanted. When the feelings of genital arousal persist for days, weeks, or even months, they are experienced as personally distressing and worrisome.

By 2007, this diagnostic criteria of PGAD had been slightly modified [2]. More emphasis was placed on the distress caused by the disorder: “genital arousal is at least moderately distressing”. Parish et al. [6] also used the original diagnostic criteria, but named a minimum time period for symptom duration of 6 months and defined possible additional symptoms that may co-occur including despair, emotional lability and suicidality.

For further differentiation, a distinction is also made between PGAD and persistent genital arousal (PGA) defined as symptoms of physiological genital arousal (e.g. genital swelling, genital sensitivity, lubrication) that occur for an extended period of time (hours or days) [7, 8] and do not meet all five of the criteria proposed by Leiblum and Nathan [1].

In 2021, a new nomenclature of PGAD was published by the International Society for the Study of Women’s Sexual Health (ISSWSH) [9]. Among others, the nomenclature of PGAD was broadened to include genito-pelvic dysesthesia (GPD) and PGAD was renamed PGAD/GPD. GPD is defined as an unpleasant, atypical sensation in the genito-pelvic region (e.g. pain, itch, vulvodynia). The change in terminology acknowledges the overlap among symptoms experienced by patients [9]. Furthermore, the ISSWSH panel recommends the following diagnostic criteria of PGAD/GPD:

1. persistent or recurrent, unwanted or intrusive, distressing sensations of genital arousal.
2. duration ≥ 3 months.
3. may include other types of genito-pelvic dysesthesia (e.g. buzzing, tingling, burning, twitching, itch, pain).
4. most commonly experienced in the clitoris but also in other genito-pelvic regions (e.g. mons pubis, vulva, vestibule, vagina, urethra, perineal region, bladder, and/or rectum).
5. may include being on the verge of orgasm, experiencing uncontrollable orgasms, and/or having an excessive number of orgasms.
6. not associated with concomitant sexual interest, thoughts, or fantasies.

PGAD is not described in the International Classification of Diseases (ICD)-10 guide [10], nor mentioned in Diagnostic and Statistical Manual of Mental Disorders (DSM)-5 [11]. It is listed in the forthcoming International Classification of Diseases (ICD)-11 [12]; however, only defined as a condition in women.

Eating disorders (ED) are among the mental disorders that are often associated with severe physical consequences and have high psychological comorbidity (DSM-5; [11]) The genesis of ED is multifactorial [13], and anorexia nervosa (AN) and bulimia nervosa (BN) predominantly affect the female gender [14].

Sexual dysfunction is common in patients with ED; nevertheless, there are few studies dedicated to this topic [15]. A scoping review failed to include any study related to sexual dysfunction in adolescent patients with AN [16]. This is noteworthy because ED often occur during puberty and adolescence, which are also sensitive periods for psychosexual development. Compared to patients with other ED, patients with AN more often report decreased sexual desire, decreased arousal, and problems achieving orgasm [16]. Malnutrition, low body mass index (BMI), and consequent secondary hypogonadism are important factors in decreased sexual desire [17]. Psychological symptoms in the context of an ED are also important cofactors for sexual dysfunction. Most notable are body dissatisfaction [18], psychological maladjustment [19], and psychological comorbidity with anxiety and depression [16]. Dunkley et al. [19] report, that women with provoked vestibulodynia (PVD) share many of the psychological features and personality characteristics which are commonly observed with an ED. They found an association of sexual pain and sexual distress with greater disordered eating.

What could be the relationship between PGAD/GPD and ED? It would be possible that one disease could cause the other, PGAD/GPD could lead to ED and vice versa. Or PGAD/GPD and ED may have a shared pathogenesis.

PGAD/GPD is a disease that creates a high level of distress and is associated with a high level of psychological comorbidity [4]. ED could be one of many possible mental health comorbidities with a shared pathogenesis or a direct result of PGAD/GPD. However, anorexia nervosa differs in a special way from other mental illnesses because anorexia nervosa directly affects the endocrine system and the capacity for genital response. AN could therefore be used as a coping strategy to prevent sexual arousal.

The close relationship from PGAD/GPD to PVD with a high prevalence of pain symptoms in PGAD/GPD [9, 20] may suggest a possible link between ED and PGAD/GPD. Dunkley et al. [19] described the link between ED symptoms and sexual pain and distress as well as the similarity of ED and PVD patient groups. Sim et al. [21] found many similarities regarding demographic, temperamental and personality characteristics of patients with ED and patients with chronic pain. In this regard, central sensitization, “a condition of over-activation of the central nervous system (CNS) that increases sensitivity to internal and external conditions” (p.14) [21], is thought to play an important role in both patient groups. It is possible that central sensitization may also be involved in PGAD/GPD and may represent the link in the pathogenesis of ED and PGAD/GPD.

In 2018, two female patients presented with PGAD/GPD in the context of an ED and were treated at the Competence Center for Eating Disorders, Parkland Clinic. Both patients provided informed consent for the publication of their medical history. A more detailed description is attached in the Addtional file 1.

### Case 1

A 15-year-old patient with AN of the restrictive type and a depressive episode reported that after menarche in the 14th year of life, genital hyperarousal occurred that lasted for hours every day. She had self-described the condition as “tingling disease” and was very ashamed of it. At that time she started to lose weight from a normal weight range to a BMI of 12 kg/m² within a few months. After having reached a significant low body weight sexual hyperarousal disappeared. AN was diagnosed and after treatment she recovered. Today the patient states that PGAD/GPD has not recurred.

### Case 2

A 38-year-old patient with a severe depressive episode with suicidality and a chronic ED reports recurrent overexcitability in the genital area, which she developed in the 14th year of life. At that time, the states of hyperarousal, which occurred several times a day, had led to the patient hardly being able to follow her school lessons. The Patient withdraw very quickly from social contacts because of strong feelings of shame. At the age of 15, she developed AN of the restrictive type and a compulsion to wash. With the development of a severe underweight condition (BMI 14 kg/m²), the symptoms of genital hyperarousal became significantly less.

At that time, the patient gained some weight during her inpatient stay, but since then, she has been chronically underweight. With gaining weight, the genital hyperarousal condition became stronger again. Today PGAD/GPD symptoms are still present, when more symptoms appear patient regulates them with losing weight.

The main objective of the systematic review is to investigate possible associations between PGAD/GPD and ED. Since PGAD/GPD is still a poorly understood disorder, a detailed overview of current studies and the clinical picture of PGAD/GPD will first be provided.

Review questions include: (1) what are the characteristics and quality of the papers found?, (2) what data on the epidemiology of PGAD/GPD are known?, (3) what sociodemographic data are known for individuals affected by PGAD/GPD?, (4) what comorbidity is known in PGAD/GPD?, (5) what is the association between ED and PGAD/GPD?, (6) what is the etiology of PGAD/GPD?, and (7) how is PGAD/GPD treated?

## Methods

This systematic review was prepared according to the Cochrane Reporting Guidelines PRISMA Statement [22].

### Systematic literature search

The systematic literature search was performed by two reviewers in the PubMed, PsycInfo, and Scopus databases, complemented by Google Scholar. In the initial literature search (search strategy 1), all studies describing PGAD/GPD in the context of ED were elicited. For this purpose, the following search terms (in title, abstract, or keywords) were used: *persistent genital arousal disorder* OR *persistent sexual arousal syndrome* OR *restless genital syndrome* combined with eating disorder* OR *anorexia* OR *bulimia* OR *binge eating* OR *OSFED* OR *EDNOS*). The syntax of this literature search is provided in Addtional file 1: Table S1. This search strategy yielded only two results via Scopus [23, 24], which, based on the titles and abstracts, did not specifically describe PGAD/GPD and ED in context.

Subsequently, it was decided to expand the search strategy and not limit it to PGAD/GPD studies related to ED (search strategy 2, for the search syntax see Addtional file 1: Table S2). The following inclusion criteria were applied: (1) studies that investigated PGAD/GPD and PGA symptomatology by December 9, 2020; (2) publication in a peer-reviewed journal; (3) study type: original study, case report, case series, review; and (4) publication language of English or German. For this literature search, titles, abstracts and keywords were searched for the following terms: *persistent genital arousal disorder* OR *persistent sexual arousal syndrome* OR *restless genital syndrome*. The search syntax is shown in the Addtional file 1. Two authors (MZ, HI) then independently screened the titles and abstracts of the records with regard to the defined inclusion and exclusion criteria.

### Selection procedure and data collection

The full texts of all retrieved studies were read through independently by three different reviewers (H.I., A.Z., S.L.). After having checked the full texts for eligibility, the following data were systematically extracted from included original studies and case reports/series: number of PGAD/GPD cases, age, sex, marital status, location, recruiting and examination, race and ethnicity, education, household income, onset of illness, proposed etiology, psychiatric history and comorbidity, treatment, PGAD/GPD criteria according to Leiblum and Nathan [1], and distress.

In regular meetings of all the authors, the results of the data collection and any inconsistencies in the extracted data were discussed until an agreement could be reached. Moreover, two researchers (M.Z., S.L.) independently rated the quality of the included cross-sectional studies using the Appraisal Tool for Cross-Sectional Studies (AXIS, [25]). Inconsistencies were discusses until consensus was reached.

## Results

### Results of the literature search

The literature search yielded 108 results in PubMed, 91 results in PsycInfo, 134 results in Scopus and 8 additional results via Google Scholar and personal contacts (total results = 341). The total number of literatures found after removing duplicates was 159; one paper full-text could not be found. After excluding book chapters, dissertations and conference papers, the titles and abstracts of 145 studies were screened for inclusion criteria. Excluded were editorials, commentaries, and letters, and publications whose publication language was not German or English, or PGAD/GPD was not the content of the study.

**Fig. 1:**
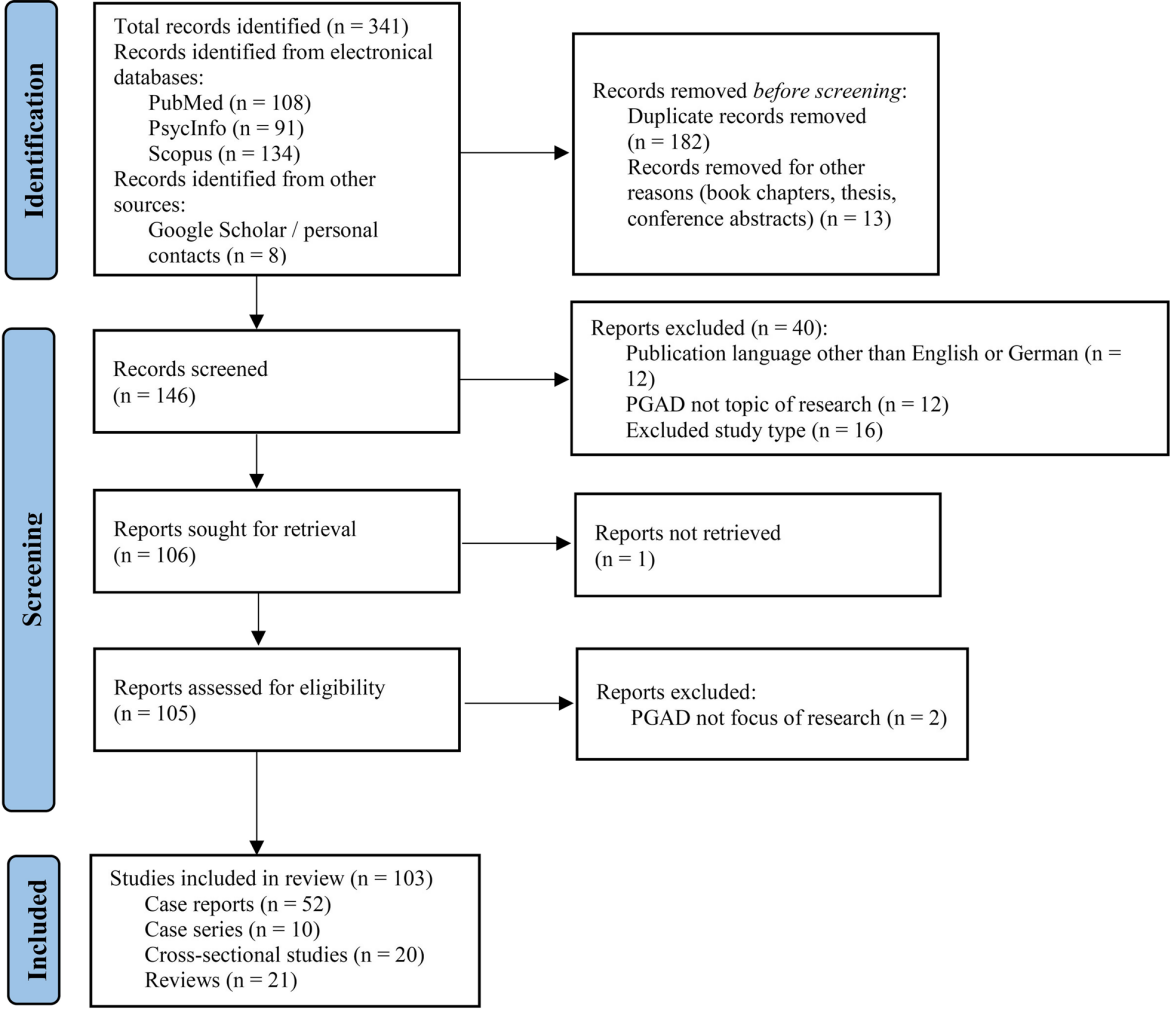
PRISMA flow chart documenting the literature search and inclusion of studies

Finally, a total of 103 studies were subsequently included in the present review (see flow chart presented in Fig. 1).

### Study situation and clinical picture of PGAD/GPD

#### Characteristics and quality of papers found

The 103 included papers comprised 52 case reports (N ≤ 2 cases), 10 case series (n > 2 cases), 20 cross-sectional studies and 21 reviews. For a detailed description of included studies see Addtional file 1: Tables S3–S6.

Data for the 52 case reports and 10 case series were collected primarily through face-to-face interviews and examinations. A total of 123 cases with PGA symptomatology were described. Eighty-one participants (66%) met all five criteria for PGAD/GPD according to Leiblum and Nathan [1]. The remaining participants met at least one PGAD/GPD criterion. For some patients, the exact number of PGAD/GPD criteria remained unclear / were not reported.

Of 20 cross-sectional studies, 14 studies were questionnaire studies or online surveys. Face-to-face interviews and examinations took place in six studies. Of all included cross-sectional studies, 2,078 cases were described as having PGA symptomatology. Of these, 892 cases met all five PGAD/GPD core criteria, and 1,186 cases met at least one PGAD/GPD criterion.

In three cross-sectional studies, patients with PGAD/GPD were compared with healthy controls [8, 26, 27]. There were four cross-sectional studies that allowed the calculation of a prevalence estimate of PGAD/GPD based on their design [28–31]. The quality ratings for the cross-sectional studies are shown in the Addtional file 1: Table S7. As most studies used a convenience sample or used online recruitment, self-selection of participants in many studies is likely which may question the representativeness of the samples. Moreover, no study so far has provided data on non-responders and only few studies were able to calculate a response rate. Some studies disclosed that non-validated measures to assess PGAD/GPD symptoms were used as such measures were simply not available. However, survey questions were often piloted prior to their use.

#### Epidemiology of PGAD/GPD

Until 2020, there were few meaningful studies on the epidemiology of PGAD/GPD [28, 29]. In 2020, two epidemiologic studies by Dèttore and Pagnini [30] and Jackowich and Pukall [31] separately assessed symptom expression using the five PGAD/GPD core criteria and associated distress.

Dèttore and Pagnini [30] studied a population of 679 female undergraduates in Florence, Italy. Eleven women (1.62%) reported the presence of all five PGAD/GPD core criteria two women (0.29%) met all five PGAD/GPD core criteria and reported maximum distress scores.

Jackowich and Pukall [31] examined two different populations. In a cohort of 1,634 undergraduates in Canada, 1.1% of males and 0.6% of females reported the presence of all five PGAD/GPD core criteria with moderate-to-high frequency. 4.5% of the participants reported moderate-to-severe distress for one or more symptoms according to the PGAD/GPD core criteria.

In a representative cohort of United States residents, of 1,026 respondents, 4.3% of men and 2.7% of women reported all five PGAD/GPD core criteria with moderate-to-high frequency. A total of 68 study participants (6.6%) reported moderate-to-severe distress for one or more symptoms according to the PGAD/GPD core criteria. In the same sample, 32.9% of respondents reported symptoms related to the first PGAD/GPD core criterion (persistent physiological sexual arousal), highlighting the high prevalence of PGA, which was consistent with the data from other studies [28, 30].

#### Sociodemographic characteristics of individuals with PGAD/GPD

Among all 2,201 PGAD/GPD cases described in case studies, case series and cross-sectional studies and who met at least one PGAD/GPD criterion, 2,167 were women and 34 were men. However, in some studies, men were excluded from participation [30].

The mean age of all described cases of PGA symptomatology in case reports and case series was 48 years (range: 16–81 years). The mean age at symptom onset in these publications was 42.5 years (range: 6–75 years).

There are few data on PGAD/GPD in adolescence. In some cross-sectional studies, adolescents were explicitly excluded from participation, and in no cross-sectional study were adolescent data separately collected. In an online survey of women with PGA symptoms, 25.2% retrospectively reported disease onset before age 18 [7]. In the case reports, there was only one description of an adolescent case (female, aged 16 years) [32]. There are other case reports in which the affected adults reported symptom onset of PGAD/GPD during adolescence [33, 34], including two women who reported onset of PGAD/GPD, at the time of menarche.

Characteristics of race and ethnicity were collected in very few papers. We, therefore, also evaluated local origin (Asia, Australia, Europe, North and South America), study location, and cross-regional recruitment, if applicable. Finance information was only collected in a few studies. Educational attainment was surveyed much more frequently in the studies, but had a wide variation in survey methods.

In a representative sample of United States residents, Jackowich and Pukall [31] found significantly higher numbers among more highly educated participants and in the non-Hispanic Black and Hispanic/Latina groups when examining the number of PGAD/GPD symptoms.

#### Comorbidity

Waldinger and Schweitzer [35] found increased comorbidity with restless leg syndrome and overactive bladder in their patients with PGAD/GPD symptoms and defined a separate clinical syndrome to be distinguished from PGAD/GPD called restless genital syndrome. Increased comorbidity with restless leg syndrome (RLS) has also been documented in other studies [7, 8, 36, 37]. Also, genital symptoms are commonly reported [7]. The frequency of clitoral and vulvar pain was reported as 35.4% and 44.3%, respectively, in two studies [7, 20]. Pukall et al. [20] grouped PGAD/GPD together with vulvodynia in a common disease category of genito-pelvic dysesthesia.

Many studies and case reports [34, 36, 38] described a high degree of psychological comorbidity in PGAD/GPD; for example, in one study, 45% of respondents reported comorbid depressive symptoms, 35% reported anxiety, and 16% reported obsessions [2]. In two studies using standardized questionnaires, patients with PGAD/GPD showed significantly more anxiety and depressive symptoms compared to a control group without PGAD/GPD symptoms [8, 27]. In this context, anxiety and depressive symptoms can occur both before and after the onset of PGAD/GPD symptomatology [7, 24]. The prevalence of suicidal ideation is strikingly common in women with PGAD/GPD [8].

Two studies have described an increased incidence of childhood sexual abuse [24, 36].

#### Relationship between ED and PGAD/GPD

Two cross-sectional studies [2, 24] investigated ED in women who met all five PGAD/GPD original criteria. In one study [24], 19.7% of respondents reported a history of an ED diagnosis. In the other study [2], 8% of respondents reported a positive history. These results were not further commented on by the authors.

In two case reports [23, 39], there were descriptions of patients with PGAD/GPD who reported a history of an ED (AN, as well as AN and BN).

No study was included that drew a connection between ED and PGAD/GPD. There was a single case report describing a current comorbid ED (BN) [40].

A few other case reports exist that describe episodes of binge eating that may be regarded as a symptom of an ED [32, 41, 42]. In the work of Zwerling et al. [42], episodes of binge eating were attributed to Kleine-Levin Syndrome.

#### Etiology of PGAD/GPD

The ideas regarding the genesis of the disease are various, and specifically, neurological, pharmacological, hormonal, vascular and psychological causes have been discussed. It has also been suspected that there are patients with PGAD/GPD for which several underlying causative factors may be simultaneously active [4, 38].

Peripheral nerve irritation is often described as a neurological cause of PGAD/GPD. Nerve irritation can be caused by small fiber sensory neuropathy [35] or triggered by mechanical compression of the peripheral nerves [43]. Meningeal cysts (Tarlov cysts), which are thought to irritate spinal nerve roots, are most commonly reported as a mechanical cause [34, 44, 45]. CNS disorders have also been described as a neurological cause of PGAD/GPD [46, 47].

The initiation or discontinuation of selective serotonin reuptake inhibitors (SSRI) or other psychotropic drugs has been cited as pharmacological causes of PGAD/GPD [29, 40, 48–52]. In an online survey, Jackowich et al. [7] found SSRI use to be the most commonly cited possible trigger for PGA symptoms. When SSRIs are discontinued, increased sensitivity of peripheral nerves [40, 49] or altered processing of sensory stimuli could occur as a rebound phenomenon [49]. Both SSRI use and SSRI discontinuation could increase genital blood flow in a hormonally mediated manner [40, 49]. However, the exact pharmacological mechanisms of action are still unknown.

The genesis of PGAD/GPD is thought to involve sex hormones. Some case reports describe the discontinuation of estrogens [32] or an estrogen-rich diet [53] as triggers for PGAD/GPD. Alteration of hormone receptors of the genital organs is suspected [53]. A change in progesterone and estradiol concentrations in the blood after menopause was considered a risk factor for PGAD/GPD by Waldinger and Schweitzer [23].

Vascular causes of PGAD/GPD have been described as pelvic varices or genital vascular changes, which can lead to peripheral nerve irritation [54–56].

Psychological causes of PGAD/GPD are discussed due to the frequent comorbidity of PGAD/GPD with mental disorders (see 3.2.4) [57]. In this context, a psychic concomitant causation is assumed; however, studies assuming a purely psychic causation of PGAD/GPD are rare. Pernot-Masson [58] describes PGAD/GPD as a form of dissociative disorder, there is one case report [59] with the same consideration.

A bio-psychosocial approach is also helpful in PGAD/GPD, since biological, psychological, and social factors influence each other in the development and maintenance of the disorder [57, 60]. How the experience of unwanted genital arousal is evaluated by the affected person seems to be particularly important. Therefore, dysfunctional attitudes toward sexuality correlate with distress from PGAD/GPD symptoms [27, 57, 61]. In addition to the cognitive and emotional appraisal of symptoms, another mechanism is likely maintaining the condition; for example, anxiety and distress can directly stimulate sexual arousal [7, 57].

Heritability of PGAD/GPD has rarely been discussed [62].

#### Treatment of PGAD/GPD

Currently, there are no Food and Drug Administration (FDA)-approved or evidence-based treatments for PGAD/GPD. All evidence regarding the treatment of PGAD/GPD derives from case reports and case series [43].

Consistent with the unclear and most likely multifactorial genesis of PGAD/GPD, a variety of therapeutic modalities have been used.

Surgical therapies include neurolysis of the pudendal nerve [43, 63], excision of Tarlov cysts [45], and clitorectomy [64]. There have been reports in which the surgical removal of Tarlov cysts resulted in complete symptom freedom in several cases [45]. Both sacral [65] and pudendal [66] neuromodulation have also been described. The use of pelvic floor physiotherapy has been reported [67] as well. The application of local anesthetics [1] or botulinum toxin [68] are other therapeutic methods.

The use of various medications to treat PGAD/GPD is common; most notably, antidepressants and anticonvulsants [51]. However, in addition to relieving symptoms, SSRIs can also trigger or exacerbate PGAD/GPD. Kruger [51] recommends anticonvulsants or the selective noradrenalin reuptake inhibitor (SNRI) duloxetine as better treatment choices.

In addition to medications, isolated reports of the use of electroconvulsive therapy (ECT) exist [39, 69].

Psychotherapy is often indicated for coping with the disease, but treatment alone rarely occurs [59, 70–72].

The therapeutic outcomes of individual treatment modalities are highly variable and inconsistent. When possible, we have included results in the tables in the Addtional file 1. To date, there is limited research on different treatment approaches and no definite recommendations on specific treatments are made.

## Discussion

### Characteristics of the papers and diagnostic criteria

The number of papers on PGAD/GPD is limited, with the majority consisting of case reports. However, a large number of patients have been included in the cross-sectional studies.

Due to the nature of recruitment, especially in regards to the many web-based studies, selection bias has to be considered. Self-selection of participants may have led to a predominance of female participants who are known to generally participate in research more often than males. There may also be the tendency that people feeling very ashamed due to possible PGAD/GPD symptoms selectively did not participate resulting in a potential underestimation of prevalence rates. Moreover, studies having recruited PGAD/GPD patients in a single medical center with a certain treatment focus may not necessarily be representative for the entire population of PGAD/GPD patients. In addition, it is likely that a relevant number of participants were recruited and/or described more than once. Thus, more multi-center and large representative general population surveys are needed to confirm and expand the current knowledge on the prevalence and symptom characteristics of PGAD/GPD.

Another major problem is that in the past, it was not possible to agree on uniform diagnostic criteria or diagnostic instruments, which leads to non-comparable prevalence data. This problem becomes evident when we compare the work of Jackowich and Pukall [31] and Dèttore and Pagnini [30], both of whom conducted the first large cohort studies. Although a very similar and aligned survey design was used in both papers, both papers drew very different conclusions from their work. Jackowich and Pukall [31] classified all respondents as PGAD/GPD cases if they met five core symptoms at a moderate-to-high frequency. Dèttore and Pagnini [30] indicated that the mere fact of meeting all five diagnostic criteria of PGAD/GPD was not sufficient to consider spontaneous and persistent genital arousal as a true disorder, a sixth criterion, “distress”, should be met. Noteworthy, apart from different case definitions used in the two aforementioned papers, the studies also used different recruitment strategies (university samples vs. representative population survey) which is also very likely to affect prevalence estimates and leading to different conclusions.

The criterion of experienced distress could indeed be an important indicator to distinguish sick individuals from healthy ones [73]. However, it remains unclear how to classify individuals who do not meet all five PGAD/GPD original diagnostic criteria and yet have moderate-to-high distress levels [2, 30].

An agreement on common diagnostic criteria could help to achieve comparable research results in the future.

### Epidemiology and sociodemographic data

The vast majority of reported PGAD/GPD cases involve women. The prevalence and expression of PGAD/GPD in male patients remains unclear and requires further investigation.

Jackowich and Pukall [31] suggested that the average number of PGAD/GPD criteria endorsed decreased with age. However, similar proportions of individuals endorsed all five Leiblum and Nathan criteria between the ages of 20 to 60.

It is striking that there are few studies of PGAD/GPD in adolescence, yet in an online survey, many affected individuals reported onset of PGAD/GPD before age 18 [7]. By limiting recruitment to adults in some studies, including epidemiologically important studies [30, 31], selection bias with regard to early-onset PGAD/GPD is suspected. As adolescence is associated with profound hormonal changes and PGAD/GPD has also been linked to hormonal dysregulations [23], inclusion of younger participants in future studies seems important. Particularly when exploring a potential association between ED and PGAD/GPD symptoms, including younger age groups is important considering the peak of ED incidence (especially anorexia nervosa) in adolescence and early adulthood.

We also suspect underreporting of PGAD/GPD during adolescence in general, as both descriptions of ED patients presented here highlight the high level of shame associated with disclosing PGAD/GPD during adolescence.

Previous data on sociodemographic distribution suggest a worldwide prevalence of PGAD/GPD. Study results by Jackowich and Pukall [31] provide preliminary evidence of a dependence of the number of reported PGAD/GPD symptoms on education and ethnicity. However, the study did not examine group differences across sociodemographic variables.

### Etiology

The etiology of PGAD/GPD remains unknown. In most described cases of PGAD/GPD, several organic and psychological genesis factors occur simultaneously. A biopsychosocial model of the disease provides the possibility to integrate different causes and influencing factors of a disease [60].

Regarding the possible genesis of PGAD/GPD, it is very often assumed that peripheral nerves are irritated by different mechanisms. Thus, the possible mechanism for the triggering of PGAD/GPD by drugs is predominantly seen in the sensitization or irritation of the peripheral nerves [40]. Tarlov cysts are also frequently discussed as a possible cause of peripheral nerve irritation [45], but they alone cannot explain the wide frequency of individual PGA symptoms in the population. Tarlov cysts occur approximately 1.5% of the population [43], in comparison to individual PGA symptoms, which occur in up to 32.9% of the population [31]. The broad spectrum of PGA symptoms in the population suggests that dysregulation of physiological regulatory processes is also active in the genesis of PGAD/GPD.

Neurophysiological processes that have a direct central effect (i.e., that influence sensory processing of genital stimuli in the CNS have received little consideration. In both descriptions of patients with an ED presented in this paper, PGAD/GPD arose in the context of menarche, suggesting an involvement of sex hormones in its genesis and supporting the considerations of Waldinger and Schweitzer [23]. Sex hormones have a direct central effect, for example, on desire and arousal [74, 75]. In addition, sex hormones affect functional connectivity and neurotransmission [76, 77] and could influence sensory processing.

Sensory processing also includes emotional appraisal of sensory stimuli. One of our ED patients also suffered from a hypersensitivity symptom, misophonia, in addition to PGAD/GPD. Misophonia is characterized by a strong negative evaluation of acoustic stimuli [78]. In misophonia, altered functional connections in CNS signal processing are described, especially in connection with emotion-processing centers [79]. The reports of central sensitization in ED also suggest an adaptive mechanism of the CNS [21]. It is possible that similar processes are involved in PGAD/GPD. Therefore, it may be important to further explore hypersensitivity symptoms of other sensory organs in patients with PGAD/GPD.

### Comorbidity and treatment

A described high comorbidity of PGAD/GPD with RLS requires further controlled investigation. Similar to PGAD/GPD, RLS occurs more frequently in menopausal women and is often associated with mental illness [80].

A high prevalence of various urogenital or pelvic symptoms in PGAD/GPD [7, 20] may be explained in part by the fact that patients with PGAD/GPD develop increased arousal sensations in the lower abdominal region. This symptomatology was included in the new nomenclature [9].

Shame regarding their own symptomatology and negative experiences with health professionals have led many women to seek treatment many years after the onset of their symptomatology [20]. This may partly explain the high comorbidity with mental illness. It also seems important to consider that psychological factors may perpetuate PGAD/GPD, but may also play a role in the genesis of the disorder. Therefore, both pharmacological and psychotherapeutic treatment of mental comorbidities seem reasonable [51, 60].

The treatment of PGAD/GPD is poorly evidence-based and further studies are needed in this area. Given the presumed multifactorial genesis and multiple comorbidities, multidisciplinary treatment is recommended.

### PGAD/GPD and ED

Overall, there is little evidence in the literature to date of a relationship between PGAD/GPD and ED. One of the reasons for this could be the lack of a systematic query of comorbid mental disorders of patients with PGAD/GPD. Moreover, in most of the studies having assessed comorbid psychopathology, the focus was on depression and anxiety but no instruments to assess ED symptoms were used. Although symptoms of depression and anxiety might be most prevalent in PGAD/GPD patients, other psychopathological symptoms have been somehow neglected in the existing research. Accordingly, depending on the focus of the study, essential information regarding the potential associations between PGAD/GPD and ED may not have been adequately captured.

In addition, we suspect the underreporting of PGAD/GPD cases during adolescence. Since many ED begin during adolescence, it is quite possible that there is also underreporting of PGAD/GPD in patients with ED. It can be assumed that PGAD/GPD symptomatology is not spontaneously reported due to high shame.

The characteristics of patients with PGAD/GPD and ED are similar in certain areas. In both diseases there is a high comorbidity with depression and anxiety, and there is a high number of reports of sexual abuse [8, 81]. ED and PGAD/GPD often affect women, onset is common in the context of hormonal changes (e.g. puberty ) [23, 82].

Dunkley et al. [19] found a positive correlation between ED symptoms and sexual dysfunction in women. In that study, psychological maladjustment acted as a mediator between the risk of developing an ED and sexual distress and pain. A connection between sexual hyperarousal and ED was not investigated. There may be a link, in that sexual pain and distress are more common in both PGAD/GPD and ED [19, 20]. Moreover, central sensitization may play a particular role in both diseases [21].

Although there is still an insufficient amount of research on the subject, we assume that there may be a link between ED and PGAD/GPD that needs further investigation. The presented cases including first descriptions of ED directly linked to PGAD/GPD provide important indications that ED may have direct and indirect influences on PGAD/GPD symptomatology. AN is a mental comorbidity that can directly reduce PGAD/GPD symptomatology via its direct influence on sex hormone secretion [17]. In addition, AN may indirectly influence PGAD/GPD symptomatology since negative emotions, especially anxiety, can trigger PGAD/GPD symptoms. AN behaviors and engagement with the ED may allow more control over negative emotions and also support emotion avoidance [83].

It is remarkable how similar the clinical picture of patients with PGAD/GPD and AN is in their attitude towards their own body. Both groups of patients strongly emotionally reject parts of their own body and react negatively to body signals from these areas. Furthermore, both patient groups try to avoid the perception of body signals. Jackowich and Pukall described hypervigilance and avoidance in an adapted fear-avoidance model to PGAD/GPD [60]. Zucker et al. [84] found enhanced subjective sensitivity to sensory experience and increased attempts to avoid sensory experiences in women with AN. This sensitivity was positively correlated with body image disturbance (e.g., feeling fat). Especially the similarity in sensory sensitivity and avoidance make AN and PGAD/GPD interesting for further investigation of possible shared underlying conditions.

Based on these considerations, patients with PGAD/GPD symptoms could represent a particularly vulnerable, high-risk group, with regard to the development of ED, especially in patients with AN.

Sexual hyperarousal should be questioned in addition to other sexual dysfunctions during the admission routine of patients with ED. We also recommend that the presence of ED in PGAD/GPD symptomatology (comorbid or in the history) should be recorded more concretely, both in clinical practice and as an implication for future research projects. Moreover, if SSRIs are used in patients with ED, it should be considered that SSRI medication can trigger PGAD/GPD symptoms.

## Conclusion

In our systematic review of the literature, we found no study that specifically investigated the co-occurrence between PGAD/GPD and ED. However, we argue that there may be several common factors that appear to be important in the etiology, course, and treatment of PGAD/PGD and AN (e.g. hormonal dysregulation, sensory sensitivity and avoidance) warranting future research on the possible comorbidity of these disorders and shared underlying conditions. AN is known to directly affect the endocrine system and the capacity for genital response. Thus, AN could therefore also be used as a coping strategy to prevent unwanted sexual arousal. Anyway, lifetime sexual dysfunction including PGAD/GPD symptoms should receive more attention in the anamnesis of patients with ED.

## Supplementary Information

**Additional file 1**. Supplementary material document.

## Data Availability

All data generated or analyzed during this study are included in this published article and its supplementary information files.

## Abbreviations

PSAS: Persistent sexual arousal syndrome
PGA: Persistent genital arousal
PGAD: Persistent genital arousal disorder
PGAD/GPD: Persistent genital arousal disorder/genito-pelvic dysesthesia
ED: Eating disorder
AN: Anorexia nervosa
BN: Bulimia nervosa
CNS: Central nervous system
SSRI: Selective serotonin reuptake inhibitors
