# Supplementary material for: Exploring the link between eating disorders and persistent genital arousal disorder/genito-pelvic dysesthesia: first description and a systematic review of the literature
Source: J Eat Disord. 2022 Nov 10;10:159. doi: 10.1186/s40337-022-00687-7 (PMC9650894; doi:10.1186/s40337-022-00687-7)
Supplement: Supplementary file 1 — Additional file 1. Supplementary material document. [file 40337_2022_687_MOESM1_ESM.docx]

***Supplementary Material***

**1 Supplementary Data**

Case 1. Full-length description of case 1

Case 2. Full-length description of case 2

**2 Supplementary Tables**

Table S1. Syntax of the original literature search (Persistent genital arousal disorder combined with eating disorders)

Table S2. Syntax of the final literature search (Persistent genital arousal disorder not limited to eating disorders)

Table S3. Case reports included in the systematic review

Table S4. Case series included in the systematic review

Table S5. Cross-sectional studies included in the systematic review

Table S6. Review articles with focus on / including information about PGAD included in the systematic review

Table S7. Quality rating of cross-sectional studies according to the AXIS appraisal tool

References

**1 Supplementary Data**

**Case 1 – Full-length description of case 1**

A 15-year-old patient with AN of the restrictive type and a depressive episode reported that after menarche in the 14th year of life, genital hyperarousal occurred that lasted for hours every day. The vulva area was constantly highly sensitive and even wearing tight clothing could trigger genital arousal, which she found extremely unpleasant. The patient reported no "orgasm equivalents", she had also never masturbated.

She had self-described the condition as "tingling disease" and was very ashamed of it. Worst of all, she said, was that she felt so abnormal. Without informing her parents, she went to the gynecologist. The gynecologist could not find any pathological findings and told her that the unwanted genital arousal was not a disease and would disappear on its own. The patient was not able to find any information about her problems on the internet and had thus labeled herself as a disabled person with a strange illness. She had first developed depressive symptoms at that time and started to lose weight from a normal weight range to a BMI of 12 kg/m² within a few months. The unwanted genital arousal ceased shortly after menstruation stopped due to the patient’s underweight status.

After having reached a significant low body weight sexual hyperarousal disappeared, but the patient developed misophonia (she had been unable to tolerate loud breathing by others in her presence).

The patient was treated in our specialized inpatient treatment program for adolescents with eating disorders after unsuccessful outpatient treatment attempts of AN. The patient mentioned her disorder for the first time in clinic in the context of an application of eye movement desensitization and reprocessing (EMDR) developed by us for patients with eating disorders. In the further course of treatment, feelings of guilt and shame were addressed in particular, as well as fears of a renewed return of the PGAD/GPD.

The patient overcame her AN. Because of a recurrence of a depressive episode, she now takes medication (an antidepressant and lamotrigine) continuously three years after treatment and is doing well with this regimen. Today the patient states that PGAD/GPD has not recurred.

**Case 2 – Full-length description of case 2**

A 38-year-old patient presents to our psychosomatic treatment center due to a severe depressive episode with suicidality and a chronic eating disorder. Treatment took place on either an inpatient and outpatient basis. During the second year of treatment, the patient reports overexcitability in the genital area, which she developed in the 14th year of life. At that time, even light touching by sitting or pressure of clothing could lead to orgasm-like states (i.e., contractions of the vagina in a state of arousal) that was perceived as extremely unpleasant. There was no history of previous illnesses or operations, and gynecological examinations had revealed no pathological findings.

The states of hyperarousal, which occurred several times a day, had led to the patient hardly being able to follow her school lessons and to withdraw very quickly from social contacts because of strong feelings of shame. At the age of 15, the patient developed AN of the restrictive type and a compulsion to wash. With the development of a severe underweight condition (BMI, 14 kg/m²), the symptoms of genital hyperarousal became significantly less. At that time, during inpatient treatment for AN, the patient confided in her therapist, who advised her to "just not think about her genital problem."

At that time, the patient gained some weight during her inpatient stay, but since then, she has been chronically underweight. With gaining weight, the genital hyperarousal condition became stronger again and she also developed a fear of wetting herself, which led to constant self-control and tension.

The patient had studied and then worked very performance-oriented in administration. Thus far, the patient has not entered into any partnerships nor experienced any forms of sexuality, not even masturbation. She has suffered from recurrent depressive episodes for several years.

The worst thing for the patient was to suffer from "a disease without a name." She performed a lot of research in the past and did not find any information regarding her symptoms. She always considered herself abnormal, "some kind of alien." PGAD/GPD symptoms are still present, when more symptoms appear patient regulates them with losing weight.

**2 Supplementary Tables**

**Table S1.** Syntax of the original literature search (Persistent genital arousal disorder combined with eating disorders)

| **Database** | **Search Syntax** | **Number of results** |
| --- | --- | --- |
| PubMed (09.12.2020) | (Persistent Genital Arousal Disorder[tw] OR Persistent Sexual Arousal Syndrome[tw] OR restless genital syndrome[tw]) AND (Eating disorder[tw] OR anorexia[tw] OR bulimia[tw] OR binge eating[tw] OR OSFED[tw] OR EDNOS[tw]) | 0 |
| PsycInfo (09.12.2020) | ((Persistent Genital Arousal Disorder OR Persistent Sexual Arousal Syndrome OR restless genital syndrome) AND (Eating disorder OR anorexia OR bulimia OR binge eating OR OSFED OR EDNOS)).mp | 0 |
| Scopus (09.12.2020) | TITLE-ABS-KEY ( ( "Persistent Genital Arousal Disorder" OR "Persistent Sexual Arousal Syndrome" OR "restless genital syndrome" ) AND (“Eating disorder” OR “anorexia” OR “bulimia” OR “binge eating” OR “OSFED” OR “EDNOS” ) ) | 2 |

**Table S2.** Syntax of the final literature search (Persistent genital arousal disorder not limited to eating disorders)

| **Database** | **Search Syntax** | **Number of results** |
| --- | --- | --- |
| PubMed (09.12.2020) | Persistent Genital Arousal Disorder[tw] OR Persistent Sexual Arousal Syndrome[tw] OR restless genital syndrome[tw] | 108 |
| PsycInfo (09.12.2020) | (Persistent Genital Arousal Disorder OR Persistent Sexual Arousal Syndrome OR restless genital syndrome).mp | 91 |
| Scopus (09.12.2020) | TITLE-ABS-KEY ("Persistent Genital Arousal Disorder" OR "Persistent Sexual Arousal Syndrome" OR "restless genital syndrome") | 134 |

**Table S3.** Case reports included in the systematic review

| **Author(s), Year** | **Age** | **Sex** | **Location** | **Age of PGAD onset** | **Proposed etiology** | **Psychiatric comorbidity & history** | **Treatment** | **Number of PGAD criteria met** | **Distress criteria considered** |
| --- | --- | --- | --- | --- | --- | --- | --- | --- | --- |
| Ahmad et al., 2018 ^[1]^ | 44 | ♀ | Pakistan | 33 | Dopaminergic System Dysregulation | None | Antifungal creams (fluconazole, ketoconazole), topical steroids (betamethasone, hydrocortisone), mefenamic acid and homeopathy practitioner with no benefit. Ropinirole with benefit. | At least 2/5 | Yes |
| Amsterdam et al., 2005 ^[2]^ | 44 | ♀ | USA | - | Soy phytoestrogens | None | Diet with limited soy products and supportive counselling | 5/5 | Yes |
| Anzellotti et al., 2010 ^[3]^ | 40 | ♀ | Italy | 39 | Functional Hyperconnectivity, epileptic focus. | None | Antidepressants (paroxetine, venlafaxine), low-dose benzodiazepines (lorazepam, diazepam, clonazepam) with no benefit, Topiramate with benefit. | 5/5 | Yes |
| Aquino et al., 2014 ^[4]^ | 65 | ♀ | USA | 62 | Parkinson´s disease, dopaminergic system dysregulation, connection with RLS. | Parkinson´s disease | Duloxetine and oxazepam with no benefit, pramipexole with benefit. | At least 1/5 | - |
| Armstrong & Vancaillie, 2016 ^[5]^ | 35 | ♀ | Australia | 20 | Bilateral pudendal neuralgia with nerve entrapment | Pelvic and genital pain | Duloxetine, amitriptyline, paracetamol, oxycodone, diazepam, surgery: bilateral pudendal nerve release, neurostimulator implantation. Combined treatment with good benefit. | 5/5 | Yes |
| Aswath et al., 2016 ^[6]^ | 40 | ♀ | India | 33 | - | None | Carbamazepine with no benefit, supportive psychotherapy with some benefit. | 5/5 | Yes |
| Battaglia & Venturoli, 2009 ^[7]^ | 29 | ♀ | Italy | 27 | Trazodone causing clitoral vascularization | Moderate depression and anxiety | Trazodone withdrawal with no benefit | 5/5 | Yes |
| Bedell et al., 2014 ^[8]^ | 51 | ♀ | USA | 51 | Periclitoral mass, compression of the dorsal nerve of the clitoris. | None | Surgery. Fully recovered | 5/5 | Yes |
| Bell et al., 2007 ^[9]^ | 57 | ♀ | United Kingdom | 2004 | Increased level of atrial natriuretic peptide due to atrial septal defect and fludrocortisone medication | None | Diazepam, gabapentin, carbamazepine, paroxetine, pregabalin, amitriptyline with no benefit. Midodrine with slight benefit. | At least 2/5 | Yes |

Table S3. (continued)

| **Author(s), Year** | **Age** | **Sex** | **Location** | **Age of PGAD onset** | **Proposed etiology** | **Psychiatric comorbidity & history** | **Treatment** | **Number of PGAD criteria met** | **Distress criteria considered** |
| --- | --- | --- | --- | --- | --- | --- | --- | --- | --- |
| Cohen, 2017 ^[10]^ | 40 | ♀ | USA | 37.5 | Pelvic trauma | Anxiety | Escitalopram, pregabalin, acupuncture, physical therapy and psychiatric treatment with no benefit, bilateral pudendal nerve block with moderate benefit. | At least 3/5 | Yes |
| Curran, 2019 ^[11]^ | 16 | ♀ | USA | - | Etonogestrel rod placement, Discontinuing SSRI | Sexual abuse in history, dysmenorrhea, depression, anxiety, obesity with frequent overeating. | Psychotherapy, resumption of SSRI, symptoms resolved. | 5/5 | Yes |
| De Magalhaes & Kumar, 2015 ^[12]^ | 57 | ♀ | United Kingdom | 57 | Cessation of SSRI (citalopram) | Depression with suicidal ideation, anxiety, binge drinking. | Psychiatric admission. Propranolol, antibiotics, vaginal pessary with no benefit, paroxetine with little benefit, benzodiazepine and duloxetine with more benefit. | 5/5 | Yes |
| Deka et al., 2015 ^[13]^ | 53 | ♀ | India | 50.5 | - | Depression | Oestrogen, cabergoline, alprazolam, vertin, cinnarizine, zolpidem, chlordiazepoxide, escitalopram, clonazepam, etizolam with no benefit. Clomipramine and Fluoxetine along with leuprolide and lignocaine gel, pelvic floor exercises with good benefit. | 4/5 | Yes |
| Dikici et al., 2015 ^[14]^ | 45 | ♂ | Turkey | 43 | - | None | Non-steroidal anti-inflammatory drugs and sertraline with no benefit, Pramipexole and gabapentin with good benefit. | 5/5 | Yes |
| Eibye & Jensen, 2014 ^[15]^ | 31 | ♀ | Denmark | 34 | Cessation of SSRI (paroxetine), Tarlov Cyst. | Anxiety, depression with suicidal ideation and somatization, dependent personality disorder, violence and sexual abuse in history, benzodiazepine abuse in history. | Psychiatric and psychotherapeutic treatment, resumption of SSRI, anxiolytic medication, physiotherapy, morphine, electroconvulsive therapy (ECT) with little benefit. | 5/5 | Yes |
|  |  |  |  |  |  |  |  |  |  |

Table S3. (continued)

| **Author(s), Year** | **Age** | **Sex** | **Location** | **Age of PGAD onset** | **Proposed etiology** | **Psychiatric comorbidity & history** | **Treatment** | **Number of PGAD criteria met** | **Distress criteria considered** |
| --- | --- | --- | --- | --- | --- | --- | --- | --- | --- |
| Elkins et al., 2014 ^[16]^ | 71 | ♀ | USA | 2011 | Dose increase of Mirapex | Parkinson’s, anxiety, depression. | Hypnotherapy with good benefit, lidocaine with no benefit. | 5/5 | Yes |
| Gadit, 2013 ^[17]^ | 54 | ♀ | USA | - | - | Anxiety, obsessive-compulsive disorder, incident of sexual abuse in history. | Citalopram, clonazepam, zopiclone with no benefit. | At least 1/5 | Yes |
| Gündüz et al., 2019 ^[18]^ | 42 | ♀ | Turkey | - | - | Anxiety, depression | Lithium, paroxetine, fluvoxamine, clomipramine, imipramine, clonazepam, amisulpride, zuclopenthixol, sertraline and quetiapine with no benefit, Benzodiazepine (diazepam) and Duloxetine with good benefit. | 5/5 | Yes |
| Hiller & Hekster, 2007 ^[19]^ | 52 | ♀ | United Kingdom | - | High anxiety levels, relationship problems combined with frequent sexual stimulation. | Anxiety | Amitriptyline, couple’s therapy with good benefit. | 5/5 | Yes |
| Hryko et al., 2017 ^[20]^ | 40 | ♀ | Poland | 19 | First sexual relationship | Personality disorder with dependent and emotionally unstable traits, tendencies toward anxiety and depression, one-month history of alcohol abuse | Psychotherapy with good benefit. Venlafaxine, sertraline, alprazolam, escitalopram and doxepin with no benefit. | 5/5 | Yes |
| Jones et al., 2016 ^[21]^ | 32 | ♀ | USA | - | Upregulation of the sensory afferent neuronal pathways | Attention deficit hyperactivity disorder, history of sexual abuse. | Topical lidocaine, clonazepam, trazodone, sertraline, pudenal blockade, pelvic floor Botox, pelvic floor therapy, psychotherapy, therapeutic yoga, animal therapy, acupuncture with no benefit, sacral neuromodulation with benefit. | 5/5 | Yes |
| Kamatchi & Ashley-Smith, 2013 ^[22]^ | 54 | ♂ | United Kingdom | 52 | Interaction between physical (neuropathic pain following vasectomy) and psychological (anxiety) factors | None | Duloxetine with no benefit, diazepam and pregabalin with benefit. | 5/5 | Yes |

Table S3. (continued)

| **Author(s), Year** | **Age** | **Sex** | **Location** | **Age of PGAD onset** | **Proposed etiology** | **Psychiatric comorbidity & history** | **Treatment** | **Number of PGAD criteria met** | **Distress criteria considered** |
| --- | --- | --- | --- | --- | --- | --- | --- | --- | --- |
| Korda et al., 2009 ^[23]^ | 46 | ♀ | USA | 14 | Hyperactive dopamine release | Depression | Morphine, enalapril, valsartan with benefit but side effects, nifedipine and carvedilol with no benefit, varenicline with benefit. | 5/5 | Yes |
| Korda et al., 2009 ^[24]^ | 52 | ♀ | Germany USA | 50 | Central serotonin deficiency after cessation of paroxetine, central hyperactive dopamine release through bipolar disorder. | Bipolar disorder | Valproic acid, phenylephrine, nortriptyline, baclofen, quetiapine with no benefit, ECT with good benefit. | 5/5 | Yes |
| Krapf & Goldstein, 2013 ^[25]^ | 25 | ♀ | USA | 24 | Labial oedema, hypermobility-type of Ehlers-Danlos syndrome | None | Sertraline with good benefit | At least 1/5 | Yes |
| Kruger & Hartmann, 2016 ^[26]^ | 39 | ♂ | Germany | 29 | Not reported | None | Antibiotics, diclofenac, pregabalin with no benefit; duloxetine with good benefit. | 5/5 | Yes |
| Leiblum & Nathan, 2002 ^[27]^ | 81 | ♀ | USA | 75 | Possible neurological abnormality | None | No data | 5/5 | Yes |
|  | 52 | ♀ | USA | 46 | Not reported | None | Depakote, Zoloft, Busbar, Prozac without benefit. | 5/5 | Yes |
|  | 51 | ♀ | Australia | - | Possible mildly tender supra-pubic sensation | None | No data | At least 1/5 | Yes |
| Mahoney & Zarate, 2007 ^[28]^ | 32 | ♀ | USA | 32 | Increased doses of venlafaxine | Bipolar disorder | Reduction of venlafaxine with good benefit | 5/5 | Yes |
| Mariyam Farzana et al., 2018 ^[29]^ | 36 | ♀ | India | - | Improper posture, myofascial trigger, overactive pelvic floor muscles. | None | Physical therapy with good benefit | 5/5 | Yes |
| McMullen & Agarwal, 2016 ^[30]^ | 29 | ♀ | USA | - | Chronic pelvic pain | Anxiety and depression; proposed bipolar disorder | Gabapentin, sertraline, duloxetine hydroxyzine; transcranial magnetic stimulation with good benefit. | 5/5 | Yes |

Table S3. (continued)

| **Author(s), Year** | **Age** | **Sex** | **Location** | **Age of PGAD onset** | **Proposed etiology** | **Psychiatric comorbidity & history** | **Treatment** | **Number of PGAD criteria met** | **Distress criteria considered** |
| --- | --- | --- | --- | --- | --- | --- | --- | --- | --- |
| Miyake et al., 2018 ^[31]^ | 65 | ♀ | Japan | 65 | Increased dose of milnacipran | Depression | Anticonvulsant gabapentin enacarbil and cessation of milnacipran with good benefit | 4/5 | Yes |
| Nazik et al., 2014 ^[32]^ | 23 | ♀ | Turkey | - | - | None | Botulinum toxin with good benefit | 5/5 | Yes |
|  | 38 | ♀ | Turkey | - | - | None | Botulinum toxin with good benefit | 5/5 | Yes |
| Philippsohn & Kruger, 2012 ^[33]^ | 36 | ♀ | Germany | 32 | Possible consequence of neuropathy | None | Paracetamol, ibuprofen, metamizole without any benefit; local anaesthetic gel with minimal benefit; duloxetine with good benefit. | 5/5 | Yes |
|  | 41 | ♀ | Germany | 41 | Starting a medication with amitriptyline | Depression | Reduction of amitriptyline without any benefit; quetiapine with benefit; pregabalin; psychodynamic psychotherapy with benefit. | 5/5 | Yes |
| Ramic, 2013 ^[34]^ | 33 | ♀ | USA | 32 | Pregnancy, neuronal dysfunction. | Anxiety | Various combinations of antidepressants and benzodiazepines, topiramate in addition to paroxetine and clonazepam with good benefit. | 5/5 | Yes |
| Rosenbaum, 2010 ^[35]^ | 27 | ♀ | Israel | 27 | Pregnancy; pelvic-related causes. | None | Physical therapy; psychoeducation with good benefit. | 5/5 | Yes |
| Sforza et al., 2017 ^[36]^ | 74 | ♀ | France | - | - | Restless legs syndrome | Clonazepam without benefit; pramipexole with good benefit. | 5/5 | Yes |
| Stevenson & Köhler, 2015 ^[37]^ | 27 | ♂ | USA | - | - | None | Physical therapy without benefit; paroxetine with good benefit. | 5/5 | Yes |
| Waldinger et al., 2010 ^[38]^ | 56 | ♀ | Nether-lands | 56 | Peripheral mononeuropathy of the pudendal nerve and the dorsal nerve of the clitoris, receptor-related causes. | None | Transcutaneous electrical nerve stimulation (TENS) with good benefit | 5/5 | Yes |
|  | 61 | ♀ | Nether-lands | 61 | Peripheral mononeuropathy of the pudendal nerve and the dorsal nerve of the clitoris | None | Vaseline lidocaine without benefit; clonazepam, oxazepam, temazepam and pregabalin without benefit; methylprednisolone and bupivacaine with temporary good benefit; transcutaneous electrical nerve stimulation with good benefit. | 5/5 | Yes |

Table S3. (continued)

| **Author(s), Year** | **Age** | **Sex** | **Location** | **Age of PGAD onset** | **Proposed etiology** | **Psychiatric comorbidity & history** | **Treatment** | **Number of PGAD criteria met** | **Distress criteria considered** |
| --- | --- | --- | --- | --- | --- | --- | --- | --- | --- |
| Waldinger & Schweitzer, 2018 ^[39]^ | 40 | ♀ | USA & Nether-lands | 40 | Cannabis and intense sexual activity | Anxiety and panic attacks | - | Not reported | Yes |
| Waldinger et al., 2010 ^[40]^ | 77 | ♀ | Nether-lands | 72 | Peripheral mononeuropathy of the pudendal nerve and the dorsal nerve of the clitoris | None | Clitoridectomy with benefit, Clonazepam with low benefit, bupivacaine with transient benefit. | 5/5 | Yes |
| Waldinger et al., 2011 ^[41]^ | 74 | ♂ | Nether-lands | 73 | - | None | TENS with good benefit | 5/5 | Yes |
|  | 38 | ♂ | Nether-lands | 34 | - | None | TENS without benefit | 5/5 | Yes |
| Wylie et al., 2006 ^[42]^ | 66 | ♀ | United Kingdom | 61 | Drowsiness and initiation of sleep | None | Physical therapy, cold compresses, distraction techniques and analgesics with little benefit; paroxetine with improvement of symptoms; carbamazepine and risperidone without benefit. | 5/5 | Yes |
| Yero et al., 2006 ^[43]^ | 52 | ♀ | USA | 49 | Start of medication with lamotrigine | Bipolar disorder | ECT with good benefit. Concomitant medication valproic acid and paroxetine with small benefit. | 5/5 | Yes |
|  | 58 | ♀ | USA | 57 | Cessation of lamotrigine and paroxetine | Bipolar disorder, Tourette syndrome. History of major depression, Anorexia nervosa and bulimia nervosa. | Sertraline with no benefit, clonazepam, topical use of lidocaine with small benefit. ECT with good benefit. | 5/5 | Yes |
| Yildirim et al., 2017 ^[44]^ | 70 | ♀ | Turkey | - | Possible hereditary basis | History of major depression | Cessation of Carbamazepine, Medication with clomipramine and quetiapine with small benefit. | 5/5 | Yes |
|  | 52 | ♀ | Turkey | 51 | Possible hereditary basis | Depression | Sertraline with no benefit, clomipramine with good benefit. | 5/5 | Yes |

Table S3. (continued)

| **Author(s), Year** | **Age** | **Sex** | **Location** | **Age of PGAD onset** | **Proposed etiology** | **Psychiatric comorbidity & history** | **Treatment** | **Number of PGAD criteria met** | **Distress criteria considered** |
| --- | --- | --- | --- | --- | --- | --- | --- | --- | --- |
| Zoorob et al., 2019 ^[45]^ | 57 | ♀ | USA | 57 | Upregulated or alternative activation of sacral nerve pathways after sacral neuromodulator placement | None | Deactivation and removal of the neuromodulator with slight benefit | At least 1/5 | Yes |
| Patricia et al., 2015 ^[46]^ | 26 | ♀ | Brasilia | 23 | Somatosensory memories of traumatic activation | Sexual abuse in early childhood, two episodes of depression. | Brainspotting with good benefit | 5/5 | Yes |
| Philippsohn, 2011 ^[47]^ | 41 | ♀ | Germany | - | Somatic and psychological factors | Depression and suicidal ideation | Acupuncture and TENS with no benefit, clonazepam plus pregabalin and psychotherapy with good benefit. | 5/5 | Yes |
|  | 36 | ♀ | Germany | 32 | - | None | Paracetamol, ibuprofen and metamizole with no benefit; topical use of lidocaine with small benefit; duloxetine with good benefit. | 4/5 | - |
| Seifert et al., 2020 ^[48]^ | 35 | ♀ | Germany | 35 | Change of medication, starting milnacipran with simultaneous dosing down of sertraline. | Borderline personality disorder, bulimia nervosa, depression. | Sertraline was increased again, resulting in a slight reduction of symptoms. | 5/5 | Yes |
| Bilal, 2020 ^[49]^ | 38 | ♀ | Pakistan | 28 | SSRI intake, relationship difficulties with partner. | Dysthymia with episodes of binge eating and constipation | Alprazolam with no benefit, antidepressants and local anaesthetic ointment with slight benefit. Mindfulness-based cognitive therapy with moderate benefit. | 5/5 | Yes |
| Ferenidou et al., 2019 ^[50]^ | 55 | ♀ | Germany | 55 | Start of medication with zolpidem | Sleep disturbance | Cessation of zolpidem, Quetiapine and olanzapine with good benefit. | 5/5 | Yes |
| Dallagiacoma, 2020 ^[51]^ | 31 | ♀ | Italy | In early child-hood | Tarlov cysts | Lifelong severe anxiety and depression, chronic tension headache and trigeminal neuralgia. | Duloxetine, referral to a specialised centre for PGAD. | At least 3/5 | - |

Table S3. (continued)

| **Author(s), Year** | **Age** | **Sex** | **Location** | **Age of PGAD onset** | **Proposed etiology** | **Psychiatric comorbidity & history** | **Treatment** | **Number of PGAD criteria met** | **Distress criteria considered** |
| --- | --- | --- | --- | --- | --- | --- | --- | --- | --- |
| Zwerling, 2020 ^[52]^ | 44 | ♀ | USA | - | Maybe related to Kleine-Levin syndrome | Comorbid Kleine-Levin syndrome with hyperphagia, posttraumatic stress disorder secondary to childhood sexual trauma, cluster headaches, major depressive disorder, anxiety and restless leg syndrome. | Treatment of PGAD symptoms with intravaginal diazepam (suppository) and topic use of lidocaine with moderate benefit. | 5/5 | Yes |

**Table S4.** Case series included in the systematic review (if data are reported case-wise in published articles, data are also reported case-wise in this table; otherwise aggregated data are presented only)

| **Author(s), Year** | **N** | | **Age [Range]** | | **Sex** | **Location** | | **Recruitment & examination** | | **Race & Ethnicity** | **Marital status** | | **Age of PGAD onset** | **Proposed etiology** | | **Psychiatric comorbidity & history** | **Treatment** | | **Number of PGAD criteria met** | | **Distress criteria considered** | |
| --- | --- | --- | --- | --- | --- | --- | --- | --- | --- | --- | --- | --- | --- | --- | --- | --- | --- | --- | --- | --- | --- | --- |
| Feigenbaum & Boone, 2015 ^[53]^ | 11 | | M=46 [33-69] | | ♀ | USA | | Patients of a larger study cohort (1045) with symptomatic meningeal cysts undergoing surgical treatment; Physical examination. | | - | - | | Not reported | Meningeal cysts in the sacral spinal canal causing sacral root compression. Tarlov cysts were found in 8 patients. | | No data | Surgical decompression of the cysts. Postoperatively, 7 patients reported complete elimination of PGAD, 3 patients had moderate benefit and 1 patient had no benefit. | | PGAD criteria met; unclear which ones. | | - | |
| Gaines et al., 2018 ^[54]^ | 4 | | M=57 [51-59] | | ♀ | USA | | Retrospective chart review of women with PGAD and implantation of a pulse generator with a lead at the pudendal nerve | | - | Married (n=3) Divorced (n=1) | | M=50 | All 4 survey respondents reported different initial triggers: initiation of bupropion therapy, motorcycle accident, after steroid injection to the pudendal nerve for pudendal neuralgia, chronic intractable yeast infection lasting more than 1 year. | | History of anxiety (n=3)  Restless leg syndrome (n=3) | Neuromodulation with placement of a tined lead at the pudendal nerve. Three patients had good benefit, and 1 patient had moderate benefit. | | 5/5 | | Yes | |
| Klifto & Dellon, 2020 ^[55]^ | 8 | | M=51 [35-64] | | ♀ | USA | | A retrospective chart review from 2010 through 2018 | | Caucasian (n=8) | Single (n=2) Married (n=5) Partner (n=1) | | - | Minimal degree of compression of the dorsal branch of the pudendal nerve after genital trauma | | Migraine  (n=4) Depression (n=3) Anxiety  (n=3) | Surgical neurolysis of the dorsal branch of the pudendal nerve. Seven of the 8 women had surgery bilaterally and reached complete remission. One woman had unilateral surgery with moderate benefit. | | 5/5 | | - | |
| Goldmeier & Leiblum, 2008 ^[56]^ | 6 | | M=48  [29-65] | | ♀ | UK | | Clinical cases | | - | Married (n=2) Widowed (n=1) No data  (n=3) | |  | Birth of the children (n=1) No data  (n=5) | |  |  | |  | |  | |
|  | |  | |  |  | |  | |  | | |  | | |  | | |  | |  | |  |

Table S4. (continued)

| **Author(s), Year** | **N** | **Age [Range]** | **Sex** | **Location** | **Recruitment & examination** | **Race & Ethnicity** | **Marital status** | **Age of PGAD onset** | **Proposed etiology** | **Psychiatric comorbidity & history** | **Treatment** | **Number of PGAD criteria met** | **Distress criteria considered** |
| --- | --- | --- | --- | --- | --- | --- | --- | --- | --- | --- | --- | --- | --- |
| Goldmeier & Leiblum, 2008 ^[56]^ (continued) |  | 29 | ♀ | UK | Physical examination | Cau-casian | - | Some years ago | - | Obsession illness, de-realization and depersonalization symptoms | Fluoxetine with no benefit | 5/5 | Yes |
|  |  | 59 | ♀ | UK | Physical examination | Cau-casian | Widowed | 58 | - | None | - | At least 2/5 | - |
|  |  | 65 | ♀ | UK | Physical examination | Cau-casian | Married | 55 | Sexual therapy,  using a vibrator. | Lifelong anorgasmia, past history of depression. | - | 5/5 | - |
|  |  | 35 | ♀ | UK | Physical examination | Cau-casian | - | 33 | - | 15-year history of severe anxiety with obsessional features | - | At least 4/5 | - |
|  |  | 52 | ♀ | UK | Physical examination | Cau-casian | - | 51 | - | - | - | At least 4/5 | Yes |
|  |  | 45 | ♀ | UK | Physical examination | Cau-casian | Married | 25 | Birth of the children | Obsessional personality and anxiety symptoms | Fluoxetine with moderate benefit | 5/5 | - |
| Leiblum & Nathan, 2001 ^[57]^ | 7 | M=46 [22-81] | ♀ | USA | Physical examination and/or Interview | - | Single (n=2) Married (n=5) |  |  | Anxiety  (n=1) Depression (n=2) No data  (n=4) |  |  | n=5 |
|  |  | 22 | ♀ | USA | Physical examination, case report from Riley (1994) | - | Single | - | Premenstrual symptoms that disappeared 24 hours after onset of the menstrual period | Comorbid anxiety, depression. | Lorazepam, oil of evening primrose, oral contraceptive without benefit, Danazol (suppressed menstruation) with good benefit. | At least 3/5 | Yes |

Table S4. (continued)

| **Author(s), Year** | **N** | **Age [Range]** | **Sex** | **Location** | **Recruitment & examination** | **Race & Ethnicity** | **Marital status** | **Age of PGAD onset** | **Proposed etiology** | **Psychiatric comorbidity & history** | **Treatment** | **Number of PGAD criteria met** | **Distress criteria considered** |
| --- | --- | --- | --- | --- | --- | --- | --- | --- | --- | --- | --- | --- | --- |
| Leiblum & Nathan, 2001 ^[57]^ (continued) |  | 36 | ♀ | USA | Interview | - | Married | 29 | - | - | 2 years of psychotherapy | At least 4/5 | Yes |
|  |  | 81 | ♀ | USA | Physical examination and  interview | - | Married | 75 | Hysterectomy | - | - | 5/5 | Yes |
|  |  | 61 | ♀ | USA | Physical examination and  interview | - | Married | 30 | Psychological factors | History of depression | 3 years of psychotherapy | 5/5 | Yes |
|  |  | 38 | ♀ | USA | Physical examination and Interview | - | Married | 38 | - | - | Oral contraceptive and lorazepam with little benefit, Topical anaesthetic with lidocaine with good benefit. | 5/5 | Yes |
|  |  | 35 | ♀ | USA | Interview | - | Single | 35 | Overstimulation of the vulva from prolonged contact with a bicycle seat. | - | - | 5/5 | - |
|  |  | 36 | ♀ | USA | Interview | - | Married | 29 | - | - | 2 years of psychotherapy | At least 4/5 | Yes |
| Oaklander et al., 2020 ^[58]^ | 10 | M=53 [29-80] | ♀ | USA | Retrospective university hospital study. Some were re-interviewed for details. | Caucasian (n=9) Arabic (n=1) | With partner  (n=6) | M=44 [11-70] | PGAD association with - Tarlov cysts (n=4) - sensory polyneuropathy (n=2)  - spina bifida occulta (n=1) - discontinuation of medication (n=1) - herniated disc (n=1) |  | Psychiatric treatment was universally ineffective.  Neurological treatments led to improvement in 4/5 participants, 2 of whom were cured. | See below | - |
|  |  | 29 | ♀ | USA | See above | Cau-casian | - | 12 | Symptomatic sacral spina bifida occulta | Depression, anxiety | Ineffective: pudendal nerve block; empiric anti-infectives for STDs, vaginal Botox injections to bladder neck, clitoris, pelvic floor, topical lidocaine. Effective: Bilateral genitofemoral nerve block gave 80% relief for 3 days only. | - | - |

Table S4. (continued)

| **Author(s), Year** | **N** | **Age [Range]** | **Sex** | **Location** | **Recruitment & examination** | **Race & Ethnicity** | **Marital status** | **Age of PGAD onset** | **Proposed etiology** | **Psychiatric comorbidity & history** | **Treatment** | **Number of PGAD criteria met** | **Distress criteria considered** |
| --- | --- | --- | --- | --- | --- | --- | --- | --- | --- | --- | --- | --- | --- |
| Oaklander et al., 2020 ^[58]^  (continued) |  | 36 | ♀ | USA | See above | Arabic | - | 32 | Current PGAD attribution:  sacral radiculopathy from multiple Tarlov cysts | Depression | Effective: Mild improvement with duloxetine, acetazolamide. Complete remission after surgical Tarlov-cyst resection | - | - |
|  |  | 37 | ♀ | USA | See above | Cau-casian | - | 11 | Small fibre neuropathy, possible plexus irritation by endometriomas | Depression, anxiety | Gradual improvement with aging | At least 1/5 | - |
|  |  | 42 | ♀ | USA | See above | Cau-casian | - | 30 | L5S1 HNP with L5 radiculopathy | Depression | Ineffective: Sex therapy; levetiracetam Effective: Mild improvement with risperidone | 5/5 | - |
|  |  | 50 | ♀ | USA | See above | Cau-casian | - | 46 | Abrupt withdrawal of medication (duloxetine) | Depression, anxiety | Remission after duloxetine administration, then tapered off for 3 weeks. | - | - |
|  |  | 58 | ♀ | USA | See above | Cau-casian | - | 58 | Sacral radiculopathy from multiple Tarlov cysts | Depression, anxiety | Ineffective: pudendal nerve blocks, caudal epidural steroids, testosterone, pelvic PT, TENS, tibial nerve stimulator, resection of Tarlov cysts. | - | - |
|  |  | 59 | ♀ | USA | See above | Cau-casian | - | 58 | - | History of depression. Current depression. | Ineffective: pudendal nerve blocks, epidural steroids, trigger point injections, gabapentin, topical amitriptyline, baclofen, local anaesthetics. Mild help from gabapentin cream; nerve blocks gave few weeks relief. | - | - |
|  |  | 61 | ♀ | USA | See above | Cau-casian | - | 53 | Atypical CIDP with small-fibre involvement plus lumbar Tarlov cysts | Depression | IVIg reduced PGAD symptoms | - | - |
|  |  | 72 | ♀ | USA | See above | Cau-casian | - | 69 | Sacral radiculopathy from right S3 Tarlov Cyst | Depression | Ineffective: bilateral pudendal genitofemoral, ilioinguinal, nerve blocks, gabapentin, pregabalin, sertraline, acupuncture, topical lidocaine, amitriptyline/gabapentin/  baclofen. | - | - |

Table S4. (continued)

| **Author(s), Year** | **N** | **Age [Range]** | **Sex** | **Location** | **Recruitment & examination** | **Race & Ethnicity** | **Marital status** | **Age of PGAD onset** | **Proposed etiology** | **Psychiatric comorbidity & history** | **Treatment** | **Number of PGAD criteria met** | **Distress criteria considered** |
| --- | --- | --- | --- | --- | --- | --- | --- | --- | --- | --- | --- | --- | --- |
| Oaklander et al., 2020 ^[58]^  (continued) |  | 80 | ♀ | USA | See above | Caucasian | - | 70 | Sacral radiculopathy from multiple Tarlov cysts | Depression, anxiety, tardive dyskinesia, benzo-diazepine dependence | Ineffective: pudendal nerve blocks, TENS, electroconvulsive therapy, Citalopram, leuprolide, Escitalopram, amitriptyline, ovarian vein embolization. | - | - |
| Leiblum & Goldmeier, 2008 ^[59]^ | 5 | M=51 [37-60] | ♀ | USA | Data from an online survey from 2005 | - | Single (n=2) Married (n=2) No data (n=1) | M=44 | See below | See below | See below | See below | See below |
|  |  | 37 | ♀ | USA | Online survey | - | Single | 28 | After start of venlafaxine intake | Chronic fatigue syndrome in history, comorbid anxiety | Escitalopram with moderate benefit | 5/5 | - |
|  |  | 52 | ♀ | USA | Online survey | - | Married | 46 | Cessation of venlafaxine | Depression in history | - | At least 3/5 | Yes |
|  |  | 60 | ♀ | USA | Online survey and telephone interview | - | Married | - | Start of fluoxetine intake | Depression in history | Change of medication to paroxetine, venlafaxine, sertraline or escitalopram with no benefit. Cessation of SSRI with good benefit. | 5/5 | Yes |
|  |  | 46 | ♀ | USA | Online survey and telephone interview | - | No data | - | Start of dose reduction of venlafaxine | Mild depression in history | Antibiotics with no effect, complete cessation of venlafaxine with good benefit. | At least 3/5 | Yes |
|  |  | 59 | ♀ | USA | Online survey | - | Single | 59 | Cessation of sertraline | - | - | At least 3/5 | - |
| Leiblum & Chivers, 2007 ^[60]^ |  | 38 | ♀ | USA | Letter | - | Married | 6 or 7 years | - | - | - | 5/5 | Yes |
|  |  | 63 | ♀ | USA | Letter | - | - | Teen-age | - | - | Sexual therapy | At least 3/5 | - |

Table S4. (continued)

| **Author(s), Year** | **N** | **Age [Range]** | **Sex** | **Location** | **Recruitment & examination** | **Race & Ethnicity** | **Marital status** | **Age of PGAD onset** | **Proposed etiology** | **Psychiatric comorbidity & history** | **Treatment** | **Number of PGAD criteria met** | **Distress criteria considered** |
| --- | --- | --- | --- | --- | --- | --- | --- | --- | --- | --- | --- | --- | --- |
| Yildirim et al., 2012 ^[61]^ | 3 | M=51 [43-61] | ♀ | Tur-key | Physical examination | - | Married (n=3) | M=48 [41-57] | See below | See below | PGAD and Islamic bathing rituals lead to a misdiagnosis of an obsessive-compulsive disorder | 5/5 | Yes (n=3) |
|  |  | 50 | ♀ | Tur-key | Physical examination | - | Married | 46 | - | Adjustment disorder with depressed mood caused by PGAD | Quetiapine, carbamazepine with no benefit; paroxetine, clomipramine, psychotherapy. | 5/5 | Yes |
|  |  | 43 | ♀ | Tur-key | Physical examination | - | Married | 41 | Son recruited for the military | Adjustment disorder with depressed mood caused by PGAD | Citalopram with no benefit, fluvoxamine with moderate benefit. Psychotherapy. | 5/5 | Yes |
|  |  | 61 | ♀ | Tur-key | Physical examination | - | Married | 57 | Son moved to a foreign country | Adjustment disorder with depressed mood caused by PGAD | Sertraline with no benefit | 5/5 | Yes |
| Yildirim et al., 2017 ^[62]^ | 7 | M=49 [26-70] | ♀ | Tur-key | Sexual Dysfunction Unit; Physical examination | - | - | M=37 [16-57] | - | See below | Clomipramine in all cases provided complete remission with a sustained effect during follow up. In all cases, 4 sessions of psychotherapy were given, and a psychoeducational follow-up was offered. | 5/5 | Yes (n=3) |
|  |  | 50 | ♀ | Tur-key | See above | - | - | 46 | - | Adjustment disorder with depressed mood | Paroxetine, quetiapine, carbamazepine, psychotherapy with no benefit; Clomipramine with good benefit. | 5/5 | Yes |
|  |  | 43 | ♀ | Tur-key | See above | - | - | 41 | - | Adjustment disorder with depressed mood | Citalopram with no benefit; fluvoxamine with moderate benefit. Clomipramine with good benefit. | 5/5 | Yes |
|  |  | 61 | ♀ | Tur-key | See above | - | - | 57 | - | Adjustment disorder with depressed mood | Sertraline carbamazepine with no benefit; Clomipramine with good benefit. | 5/5 | Yes |

Table S4. (continued)

| **Author(s), Year** | **N** | **Age [Range]** | **Sex** | **Location** | **Recruitment & examination** | **Race & Ethnicity** | **Marital status** | **Age of PGAD onset** | **Proposed etiology** | **Psychiatric comorbidity & history** | **Treatment** | **Number of PGAD criteria met** | **Distress criteria considered** |
| --- | --- | --- | --- | --- | --- | --- | --- | --- | --- | --- | --- | --- | --- |
| Yildirim et al., 2017 ^[62]^  (continued) |  | 40 | ♀ | Tur-key | See above | - | - | 16 | - | Mild depressive symptoms, obsessive compulsive symptoms | Sertraline, mirtazapine, fluvoxamine, ziprasidone with no benefit. Clomipramine with good benefit. | 5/5 | - |
|  |  | 26 | ♀ | Tur-key | See above | - | - | 20 | - | Bipolar depression in remission | Sertraline with no benefit. Clomipramine with good benefit. | 5/5 | - |
|  |  | 70 | ♀ | Tur-key | See above | - | - | 26 | - | Major depressive disorder, psychogenic non-epileptic seizures. | Carbamazepine, diazepam, sertraline with no benefit. Clomipramine with good benefit. | 5/5 | - |
|  |  | 52 | ♀ | Tur-key | See above | - | - | 51 | - | Adjustment disorder with depressed mood | Sertraline with no benefit. Clomipramine with good benefit. | 5/5 | - |

**Table S5.** Cross-sectional studies included in the systematic review

| **Author(s), Year** | **N** | **Age** | **Sex** | **Location** | **Recruit-ment & exami-nation** | **Age of PGAD onset** | **Race & Ethnicity** | **Marital status** | **Education** | **Proposed etiology** | **Psychiatric comorbidity & history** | **Treat-ment** | **Number of PGAD criteria met** | **Distress criteria considered** |
| --- | --- | --- | --- | --- | --- | --- | --- | --- | --- | --- | --- | --- | --- | --- |
| Carvalho et al., 2013 ^[63]^ | 43 with PGAD | M=48.5 Exclusion:  <18 years | ♀ | Portugal North American (69.9%), Central American (1.4%), South American (0%),  European (24.7%), Australian (4.1%), African (0%). | Online survey | - |  | Single (37.2%),  Married (41.9%), Divorced (16.3%), Widowed (0%), Living together (2.3%), Separated (2.3%). | 0-4 years (4.7%), 5-6 years (0%), 7-9 years (0%), 10-12 years (25.6%), 13-15 years (25.6%), 15+ years (44.2%). | - | - | - | 5/5 | Yes, but was not recorded separately but included in the 5^th^ criterion |
| Carvalho et al., 2015 ^[64]^ | 117 Participants who fulfilled some or all of the PGAD symptoms | M=36.38 Exclusion: <18 years | ♀ | Portugal North American (47.4%), Central American (0.9%), South American (1.8%),  European (45.6%), Australian (0.9%), African (3.5%). | Online survey | - |  | Single (56.4%),  Married (23.1%), Divorced (6.8%), Widowed (0.9%), Living together (10.3%), Separated (2.6%). | 0-4 years (4.3%),  5-6 years (1.7%),  7-9 years (0.9%),  10-12 years (12%), 13-15 years (30.8%), +15 years (50.4%). | - | - | - | Participants answered a checklist that recorded the frequency of the first 4 criteria and, in addition, the associated level of distress. | - |
| Garvey et al., 2008 ^[65]^ | n= 96,  1 with PGAD,  32 with spontaneous and PGAD symptoms | M=28 (18 to 64 years) | ♀ | UK | Sexual Dysfunction Unit, Physical exa-mination | - | - | Single (44.8%), Partner (49%). | Up to 18 years (75%) | - | Depression, anxiety, panic, history of sexual assault | Medication | 5/5 | Yes |
| Healy et al., 2018 ^[66]^ | n=300, 6 with PGAD | Range: 15 to 66 years | ♀: 4 ♂: 2  With PGAD | UK, Canada | Online survey | - |  | - | - | Medication (SSRI) | - | - | - | - |

Table S5. (continued)

| **Author(s), Year** | **N** | **Age** | **Sex** | **Location** | **Recruit-ment & exami-nation** | **Age of PGAD onset** | **Race & Ethnicity** | **Marital status** | **Education** | **Proposed etiology** | **Psychiatric comorbidity & history** | **Treat-ment** | **Number of PGAD criteria met** | **Distress criteria considered** |
| --- | --- | --- | --- | --- | --- | --- | --- | --- | --- | --- | --- | --- | --- | --- |
| Jackowich et al., 2018b ^[67]^ | 55 with PGAD**,** 60 fulfilled individual symptoms of PGAD | M=46 (18 to 79 years) | ♀ | Canada, Canadian (10.4%), US-American (55.7%), Western European (13%), Other (17.4%). | Online survey | M= 37.05 (6 to 66 years) |  | Single (30.4%), Dating (10.4%), Partner/married (59.1%). | College degree (42.6%), Professional degree (41.7%),  High school (12.2%). | - | Posttraumatic stress disorder (13.9%) | - | 5/5 | Was surveyed separately |
| Jackowich et al., 2018 ^[68]^ | 42 with PGAD Symptoms,  37 with painful PGAD Symptoms, 42 with Chronic Vulvar Pain | M=46 | ♀ | Subset of previous survey Canadian (11.9%), US-American (52.4%), Western European (19%),  Other (16.7%). | Online survey | M= 38.1 |  | Single (35.7%), Dating (7.1%), Partner/married (57.1%). | College degree (42.9%), Professional degree (50%), High school (7.1%). | - | Impaired daily functioning (FSQ) | - | At least 1/5 | - |
| Jackowich et al., 2020 ^[69]^ | 72 with PGAD vs. 72 non PGAD | M=45.4 | ♀ | Subset of previous survey, Canadian (6.9%), US-American (55.6%), Western European (18.1%), Other (11.1%). | Online survey | - |  | Single (29.2%), Dating (11.1%), Partner/married (59.7%). | College degree (41.7%),  Professional degree (44.4%), High school (11.1%). | - | Depression, suicidality, anxiety. | - | At least 1/5 | - |
| Komisaruk & Lee, 2012 ^[70]^ | 18 with PGAD | - | ♀ | USA | Web-based MRI study | - | - | - | - | Tarlov´s cysts | - | None | 5/5 | Yes |
| Leiblum et al., 2005 ^[71]^ | n = 103; 55 with PGAD (53%); 98% have fulfilled at least 1 PGAD criterion | M=39 | ♀ | USA, worldwide | Online survey | - | - | Married (39.8%) | M = 14.5 years | - | Depressed mood (42.7%), Anxiety/ panic attacks (31.1%), Obsessive compulsive symptoms (22.3%). | - | 5/5 | 25% low  35% medium 40% high |

Table S5. (continued)

| **Author(s), Year** | **N** | **Age** | **Sex** | **Location** | **Recruit-ment & exami-nation** | | **Age of PGAD onset** | **Race & Ethnicity** | **Marital status** | **Education** | **Proposed etiology** | **Psychiatric comorbidity & history** | **Treat-ment** | **Number of PGAD criteria met** | **Distress criteria considered** |
| --- | --- | --- | --- | --- | --- | --- | --- | --- | --- | --- | --- | --- | --- | --- | --- |
| Leiblum et al. 2007 ^[72]^ | n = 382;  206 with PGAD, 176 non-PGAD | PGAD:  M=38.0 non-PGAD:  M=35.2 | ♀ | USA | Online survey | | - | - | PGAD:  Married (51.9%),  Single (35.9%), Divorced or widowed (12.1%).  non-PGAD:  Married (44.6%),  Single (43.4%), Divorced or widowed (12%). | PGAD:  M=15.1 years non-PGAD: M=14.4 years | - | PGAD: eating disorders (8%), Depression (45%), Anxiety (35%), Obsessive compulsive symptoms (24%).  non PGAD: eating disorders (5%),  Depression (34%),  Anxiety (11%),  Obsessive compulsive symptoms (16%). | - | 5/5 | Yes |
| Leiblum et al., 2007b ^[73]^ | n = 124, 76 with PGAD,  48 non-PGAD (met only some criteria) | PGAD:  M=42 non-PGAD:  M=42 | ♀ | USA | | Online survey | - | - | PGAD: average 16-year partnership non-PGAD: average 16-year partnership | PGAD: M=15 years non-PGAD: M=15 years | PGAD: Sexual abuse contributed to PGAD in 11.8%.  non-PGAD: In 16%, sexual abuse contributed to PGAD. | PGAD: Anxiety (42.1%) Panic attacks (31.6%), Eating disorders (19.7%), Depression (57.9%). non-PGAD:  Anxiety (27.1%), Panic attacks (14.6%), Eating disorders (8.3%), Depression (39.6%). | - | 5/5 | Yes PGAD: 100% moderate or great distress  non-PGAD:  58% moderate or great distress |
| Leiblum et al., 2009 ^[74]^ | 172 with PGAD | M=40 | ♀ | USA | | Online survey | - | - | Married (72.1%) | High school education or equivalent: 22% | - | Depression (45.93%), Anxiety (34.3%). | None | 5/5 | Yes |
|  |  |  |  |  | |  |  |  |  |  |  |  |  |  |  |

Table S5. (continued)

| **Author(s), Year** | **N** | **Age** | **Sex** | **Location** | **Recruit-ment & exami-nation** | | **Age of PGAD onset** | **Race & Ethnicity** | **Marital status** | **Education** | **Proposed etiology** | **Psychiatric comorbidity & history** | **Treat-ment** | **Number of PGAD criteria met** | **Distress criteria considered** |
| --- | --- | --- | --- | --- | --- | --- | --- | --- | --- | --- | --- | --- | --- | --- | --- |
| Pink et al., 2014 ^[75]^ | 15 with PGAD | M= 7.9 | ♀ | Canada (Pain Management Clinic in Toronto /Quebec) | Inter-view | | M= 25,6  (4 to 57 years) | - | 60% in a relationship, on average 12 years | - | PGAD was directly related to Sexual abuse (n=1); Surgery to remove scar tissue around ureter (n=1); Injection into buttocks (n = 1); Accidental transection of pudendal nerve (n=1). | Depression (60%), Anxiety (40%), History of sexual abuse (46.7%). | Duloxetine with benefit (n = 1) Topical EMLA cream with benefit (n = 1) | 5/5 | No data |
| Squibb et al., 2019 ^[76]^ | 51 with PGAD | M=47  (21 to 47 years) | ♀  (recruited fe-males exclusievely) | USA  US-American (88.2%),  European (11.8%) | | Participants contacted via email, 2 samples (Center for Pain Medicine and Facebook support group) Questionnaire survey | - | Caucasian (92.2%), Black or African American (3.9%),  Multiracial (2%) Other (2%) | Single (15.7%),  Married (62.7%),  Separated (2%), Divorced (11.8%), Partnership (7.8%). | - | Initiation, change, or discontinuation of antidepressant therapy | - | - | PGAD criteria according to Waldinger and Schweitzer (2009)  5/5 | Surveyed separately with questionnaire (Female Sexual Distress Scale) |
| 15. Villot et al., 2016 ^[77]^ | 23 with PGAD | M=55.5  (39 to 64 years) | ♀ | France | | Department of Neurology; Medical interview and complete neurological examination | - | - | - | - | Pudendal neuropathy  But no conclusion possible because control group was missing. | Depression (13%), Associated Perineal pain (69.5%),  Overactive bladder (30.4%),  Restless legs (8.7%). | - | 5 PGAD criteria according to Basson et al. (2004)  5/5 | - |
|  |  |  |  |  | |  |  |  |  |  |  |  |  |  |  |

Table S5. (continued)

| **Author(s), Year** | **N** | **Age** | **Sex** | **Location** | **Recruit-ment & exami-nation** | **Age of PGAD onset** | **Race & Ethnicity** | **Marital status** | **Education** | **Proposed etiology** | **Psychiatric comorbidity & history** | **Treatment** | **Number of PGAD criteria met** | **Distress criteria considered** |
| --- | --- | --- | --- | --- | --- | --- | --- | --- | --- | --- | --- | --- | --- | --- |
| Waldinger & Schweitzer, 2009 ^[78]^ and Waldinger et al., 2009b ^[79]^ | 18 with PGAD | M= 53.7 | ♀ | Nether-lands | Department of Neuro-sexology, Netherlands Referral by general practitioner, gynaecologist or sexual health practitioner interview, laboratory and examination (MRI of brain and pelvis) in department | M=49.5   In the majority, onset was after/during menopause | - | Partnership (83%), of which 80% were married,  divorced (n=1) | Lower vocational education (11%) Intermediate vocational education (33%) Higher vocational education (11%) Lower general secondary education (28%)  Higher general secondary education (17%) | Cessation of SSRI (n=1), After consumption of marijuana (n=1), After Cessation of marijuana medication (n=1); Otherwise strong association with restless legs/ overactive bladder. | In history but not related to PGAD:  Depression (n=1), Anorexia nervosa (22-23 years) (n=1),  Burnout (n=1), adjustment disorder (n=1), Periods of alcohol abuse during psychosocial stress (28%), Alcohol abuse (n=1),  Childhood sexual abuse (17%). | No treatment (61.1%) Pre-treatments: Cyproterone, Psychotherapy, Pelvic floor muscle exercise (PME), haptonomy, diazepam, acupuncture. In the case of one person, treatment with clonazepam with benefit. | 5 PGAD criteria according to Basson et al. (2003)   5/5 | - |
| Waldinger et al., 2009c ^[80]^ | 23 with PGAD | M= 54.3  (32 to 77 years) | ♀ | Nether-lands | Department of Neurosexology, Netherlands Referral by general practitioner, gynaecologist or sexual health practitioner  Interviews, routine and laboratory tests, electroencephalograms, MRI of brain and pelvis | M=51.1 | - | - | Lower vocational education (17%) Intermediate vocational education (22%) Higher vocational education (35%) Lower general secondary education (13%) Higher general secondary education (9%) University education (4%) | Cessation of SSRI (n=2), After consump-tion of mariju-ana or cessation of marijuana medication (n=2), Norethisterone treatment & after ganglion stellatum blockade (n=2), Pelvic varices play an important role in the pathogenesis of ReGS. | In history but not related to PGAD: Depression (n=1), Anorexia nervosa at a young age (n=1), Burnout (n=1), adjustment disorder (n=1), Alcohol abuse (n=1),  Childhood sexual abuse (n=2). | 1 ml bupivacaine hydrochloride monohydrate 0.5% was tested: complete disappearance of ReGS for 12-96 hours, but symptoms returned completely thereafter (34.8%), no effect (13%). | 5 PGAD criteria according to Basson et al. (2003)  5/5 | Yes |

Table S5. (continued)

| **Author(s), Year** | **N** | **Age** | **Sex** | **Location** | **Recruit-ment & exami-nation** | **Age of PGAD onset** | **Race & Ethnicity** | **Marital status** | **Education** | **Proposed etiology** | **Psychiatric comorbidity & history** | **Treatment** | **Number of PGAD criteria met** | **Distress criteria considered** |
| --- | --- | --- | --- | --- | --- | --- | --- | --- | --- | --- | --- | --- | --- | --- |
| Jackowich & Pukall, 2020 ^[81]^ | Study 1:  n=1634 11 with PGAD Study 2:  n=1026  36 with PGAD | Mean age all partici-pants Study 1: M=18.15  Study 2:  M=46.1 | All participants: Study 1: ♀: 1267 ♂ = 360 diverse: = 7  Study 2:  ♀: 514 ♂ =506 diverse = 6  PGAD patients:  Study 1: ♀: 7 ♂: 4  Study 2:  ♀: 14 ♂: 22 | Study 1:  Canada Study 2:  USA | Online survey | - | Study 2: non-Hispanic white (61.8%);  non-Hispanic black (12%);  Hispanic/Latin (17.5%);  Asian (5.5%);  Native American (0.7%) | - | Study 2: Less than a high school diploma (13.5%), High school diploma (27.7%), Some college (20.8%), Associate degree (7.8%), Bachelor's degree (18.7%), Graduate degree (11.6%). | - | Restless-leg syndrome, irritable bowel syndrome, Chronic pelvic pain, Fibromyalgia. | - | 5/5 | - |
| Dettore & Pagnini, 2020 ^[82]^ | n=263;  11 PGAD  at least 1 PGAD criterion:  n=252 | Means age all partici-pants M=21.36  (18 to 46)  Exclusion: <18 years | ♀ Exclusively female participants recruited | Italy | University of Florence Questionnaires; no personal examina-tion | - | - | - | Data for all participants  13 years (78.2%), 16 years (18.11%), 18 years (1.91%), No data (1.7%). | - | - | - | 5/5 | - |

**Table S6.** Review articles with focus on / including information about PGAD included in the systematic review

| **Author(s), Year** | **Commentary** |
| --- | --- |
| Bronner et al., 2017 ^[83]^ | There are four common types of sexual preoccupation behaviour (SPB) in people with Parkinson´s disease: sexual behaviour with underlying sexual dysfunction, sexual desire discrepancy with a partner after restored desire, hypersexuality and compulsive sexual behaviour and sexual behaviour with underlying restless genital syndrome (ReGS)/ Persistent Genital Arousal Disorder (PGAD). Therapy recommendations are provided for all SPBs. |
| Brotto et al., 2010 ^[84]^ | Review of literature from 2003 to 2010 on female sexual dysfunction, regarding diagnosis, pathophysiology, investigation and treatment. Small section with PGAD with treatment recommendations. |
| Dick, 2021 ^[85]^ | Literature review regarding botulinum neurotoxin as treatment for female sexual dysfunction: botulinum neurotoxin can reduce dyspareunia and vaginismus, clinical improvement of two PGAD cases by injection of botulinum neurotoxin. |
| Facelle et al., 2013 ^[86]^ | Literature review on PGAD studied published in PubMed until 2012, summary of possible causes and treatments. |
| Giraldi et al., 2013 ^[87]^ | Diagnosis and treatment of Female Sexual Arousal Disorder. Brief summary of PGAD/ReGS literature, aetiology, symptoms and treatment. |
| Goldmeier & Leiblum, 2006 ^[88]^ | Brief review of persistent genital arousal syndrome and description of persistent sexual arousal syndrome (PSAS). |
| Goldmeier et al., 2009 ^[89]^ | Review of literature and treatment recommendations; addition of 6th diagnostic criterion ‘distress’. |
| Higgins et al., 2010 ^[90]^ | Overview of incidence, possible pathophysiology and treatment of sexual dysfunction triggered by treatment with antidepressants. PGAD is mentioned only in marginal terms. |
| Jackowich et al., 2016 ^[91]^ | Comprehensive overview of conceptualization, possible causes, impact and treatment of PGAD; literature review through 2016. Tables on individual case reports. |
| Kingsberg et al., 2017 ^[92]^ | Global overview of the literature on female sexual dysfunction regarding diagnosis and therapy. Only few comments on PGAD; the lack of data is mentioned. |
| Klifto & Dellon, 2019 ^[93]^ | Summary of the anatomy, physiology, aetiology, diagnosis and treatment of the relevant peripheral nerves involved in PGAD pathology. Nerve entrapment may be a source of constant arousal, chirurgical intervention may be curative. |
| Kruger, 2018 ^[94]^ | Reports on case reports and surveys on medications that can trigger, relieve or even worsen PGAD. SSRIs can also lead to other sexual dysfunctions; anticonvulsants or duloxetine may be better choices. |
| Markos & Dinsmore, 2013 ^[95]^ | Similarities between PGAD, ReGS and vulvodynia. PGAD and ReGS are considered subtypes of vulvodynia. |
| Parish et al., 2016 ^[96]^ | Development of a nomenclature with classification systems for disorders of female sexual desire, arousal and orgasm disorders. New nomenclature for PGAD. |
| Pernot-Masson, 2020 ^[97]^ | Overview of studies published in English on PGAD and sexual abuse/dissociative disorders. Hypothesis that PGAD is a dissociative disorder. |
| Pukall et al., 2019 ^[98]^ | Review of the literature on PGAD with a focus on the presence of pain symptoms; relationship between pain and arousal not yet adequately explored, but important. PGAD should be conceptualized as a sensory disorder to reduce stigma. |
| Schenck et al., 2007 ^[99]^ | Comprehensive review of a broad spectrum of sleep-related disorders associated with abnormal sexual behaviour. A case report of sleep exacerbation of PSAS in a postmenopausal woman. |
| Towe et al., 2020 ^[100]^ | Compilation of literature on nutrition and sexual dysfunction in women. Obesity is associated with sexual dysfunction. In women with eating disorders, sexual dysfunction occurs more frequently due to psychiatric comorbidity and emaciation-induced hypogonadism. Possible persitent sexual arousal only mentioned in marginal terms. |
| Yafi et al., 2015 ^[101]^ | Review of current literature on penile priapism, clitoral priapism and PGAD. |
| Basson, 2004 ^[102]^ | Revision of the definitions of female sexual dysfunction. Previous model is based on genital-focused events. Elaboration of an alternative model that reflects other reasons/incentives women have for sexual activity. Illustration of a diagnostic algorithm for the diagnosis of sexual dysfunction. |
| Jackowich & Pukall, 2020 ^[103]^ | PGAD has a multifactorial aetiology: biological and neurological factors as well as psychosocial factors (cognitive factors, affective factors, personality factors and sexual abuse). Psychosocial consequences are depression, anxiety and sexual relational ability. Application of the Fear-Avoidance Model to PGAD. |

**Table S7.** Quality rating of cross-sectional studies according to the AXIS appraisal tool

| Study | 1 | 2 | 3 | 4 | 5 | 6 | 7 | 8 | 9 | 10 | 11 | 12 | 13 | 14 | 15 | 16 | 17 | 18 | 19 | 20 |
| --- | --- | --- | --- | --- | --- | --- | --- | --- | --- | --- | --- | --- | --- | --- | --- | --- | --- | --- | --- | --- |
| Carvalho et al., 2013 ^[63]^ | 1 | 1 | 2 | 1 | 1 | 2 | 2 | 1 | 1 | 1 | 1 | 2 | ? | 2 | 1 | 1 | 1 | 1 | 2 | 1 |
| Carvalho et al., 2015 ^[64]^ | 1 | 1 | 2 | 1 | 1 | 2 | 2 | 1 | 1 | 1 | 1 | 2 | ? | 2 | 1 | 1 | 1 | 1 | ? | 1 |
| Garvey et al., 2008 ^[65]^ | 1 | 1 | 2 | 1 | 1 | 1 | 2 | 1 | 2 | 2 | 1 | 2 | 1 | 2 | 1 | 1 | 1 | 1 | ? | 1 |
| Healy et al., 2018 ^[66]^ | 2 | 1 | 2 | 2 | ? | 2 | 2 | ? | 2 | 2 | 2 | 2 | ? | 2 | 1 | 1 | 1 | 2 | 2 | 2 |
| Jackowich et al., 2018b ^[67]^ | 1 | 1 | 2 | 1 | 1 | 2 | 2 | 1 | 1 | 1 | 1 | 1 | 1 | 2 | 1 | 1 | 1 | 1 | 2 | 1 |
| Jackowich et al., 2018 ^[68]^ | 1 | 1 | 2 | 1 | 1 | 2 | 2 | 1 | 1 | 1 | 1 | 1 | ? | 2 | 1 | 1 | 1 | 1 | 2 | 1 |
| Jackowich et al., 2020 ^[69]^ | 1 | 1 | 2 | 1 | 1 | 2 | 2 | 1 | 1 | 1 | 1 | 1 | ? | 2 | 1 | 1 | 1 | 1 | 2 | 1 |
| Komisaruk & Lee, 2012 ^[70]^ | 1 | 2 | 2 | 2 | 2 | 2 | 2 | 1 | 1 | 2 | ? | 2 | 1 | 2 | 1 | 1 | 1 | 1 | 2 | 1 |
| Leiblum et al., 2005 ^[71]^ | 1 | 1 | 2 | 1 | 1 | 2 | 2 | 1 | 1 | 1 | 1 | 1 | ? | 2 | 1 | 1 | 1 | 1 | ? | 1 |
| Leiblum et al. 2007 ^[72]^ | 1 | 1 | 2 | 1 | 1 | 2 | 2 | 1 | 1 | 1 | 1 | 1 | ? | 2 | 1 | 1 | 1 | 2 | 2 | 1 |
| Leiblum et al., 2007b ^[73]^ | 1 | 1 | 2 | 1 | 1 | 2 | 2 | 1 | 2 | 1 | 1 | 1 | ? | 2 | 1 | 1 | 1 | 1 | 2 | 1 |
| Leiblum et al., 2009 ^[74]^ | 1 | 1 | 2 | 1 | 1 | 2 | 2 | 1 | 1 | 1 | 1 | 1 | ? | 2 | 1 | 1 | 1 | 1 | 2 | 1 |
| Pink et al., 2014 ^[75]^ | 1 | 1 | 2 | 1 | 1 | 2 | na | 1 | 2 | 2 | 1 | 1 | 2 | na | 1 | 1 | 1 | 1 | ? | 1 |
| Squibb et al., 2019 ^[76]^ | 1 | 1 | 1 | 1 | 1 | 2 | 2 | 1 | 1 | 1 | 1 | 1 | 1 | 2 | 1 | 1 | 1 | 1 | 2 | 1 |
| Villot et al., 2016 ^[77]^ | 2 | 1 | 2 | 2 | 2 | 2 | 2 | 1 | 1 | 2 | 1 | 1 | ? | 2 | 1 | 1 | 1 | 2 | ? | 1 |
| Waldinger & Schweitzer, 2009 ^[78]^ | 2 | 1 | 2 | 1 | 1 | 1 | na | 1 | 1 | 2 | 1 | 1 | 2 | na | 1 | 1 | 1 | 1 | 2 | 1 |
| Waldinger et al., 2009b ^[79]^ | 2 | 1 | 2 | 1 | 1 | 1 | na | 1 | 1 | 1 | 1 | 1 | 2 | na | 1 | 1 | 1 | 1 | 2 | 1 |
| Waldinger et al., 2009c ^[80]^ | 1 | 1 | 2 | 1 | 1 | 1 | na | 1 | 1 | 1 | 1 | 1 | 2 | na | 1 | 1 | 1 | 1 | 2 | 1 |
| Jackowich & Pukall, 2020 ^[81]^ | 1 | 1 | 1 | 1 | 1 | 2/1* | 2 | 1 | 1 | 1 | 1 | 2 | 2 | 2 | 1 | 1 | 1 | 1 | 2 | 1 |
| Dettore & Pagnini, 2020 ^[82]^ | 1 | 1 | 2 | 1 | 1 | 2 | 2 | 1 | 1 | 1 | 1 | 2 | ? | 2 | 1 | 1 | 1 | 1 | 2 | 1 |

*Appraisal questions*: 1. Were the aims/objectives of the study clear?, 2. Was the study design appropriate for the stated aim(s)? 3. Was the sample size justified? 4. Was the target/reference population clearly defined?, 5. Was the sample frame taken from an appropriate population base so that it closely represented the target/reference population under investigation?, 6. Was the selection process likely to select subjects/participants that were representative of the target/reference population under investigation?, 7. Were measures undertaken to address and categorize non-responders?, 8. Were the risk factor and outcome variables measured appropriate to the aims of the study?, 9. Were the risk factor and outcome variables measured correctly using instruments/measurements that had been trialed, piloted or published previously?, 10. Is it clear what was used to determined statistical significance and/or precision estimates? (e.g. p-values, confidence intervals), 11. Were the methods (including statistical methods) sufficiently described to enable them to be repeated?, 12. Were the basic data adequately described?, 13. Does the response rate raise concerns about non-response bias?, 14. If appropriate, was information about non-responders described?, 15. Were the results internally consistent?, 16. Were the results presented for all the analyses described in the methods?, 17. Were the authors' discussions and conclusions justified by the results?, 18. Were the limitations of the study discussed?, 19. Were there any funding sources or conflicts of interest that may affect the authors’ interpretation of the results?, 20. Was ethical approval or consent of participants attained?
*Coding:* 1 = yes, 2 = no, ? = unclear (cannot be evaluated), na = not applicable
* results of two studies are presented in this paper

**References**

[1] Ahmad, I., Rashid, S., & Rathore, F. A. (2018). Restless Genital Syndrome: Case Report of a Rare Disorder from Pakistan. *Cureus, 10*(5), e2619. <https://doi.org/10.7759/cureus.2619>

[2] Amsterdam, A., Abu-Rustum, N., Carter, J., & Krychman, M. (2005). Persistent sexual arousal syndrome associated with increased soy intake. *The Journal of Sexual Medicine*, *2*(3), 338–340. <https://doi.org/10.1111/j.1743-6109.2005.20358.x>

[3] Anzellotti, F., Franciotti, R., Bonanni, L., Tamburro, G., Perrucci, M. G., Thomas, A., … Onofrj, M. (2010). Persistent genital arousal disorder associated with functional hyperconnectivity of an epileptic focus. *Neuroscience*, *167*(1), 88–96. <https://doi.org/10.1016/j.neuroscience.2010.01.050>

[4] Aquino, C. C., Mestre, T., & Lang, A. E. (2014). Restless genital syndrome in Parkinson disease. *JAMA Neurology*, *71*(12), 1559–1561. <https://doi.org/10.1001/jamaneurol.2014.1326>

[5] Armstrong, G. L., & Vancaillie, T. G. (2016). Combined site-specific sacral neuromodulation and pudendal nerve release surgery in a patient with interstitial cystitis and persistent arousal. *BMJ Case Reports*, *2016*, bcr2015213513. <https://doi.org/10.1136/bcr-2015-213513>

[6] Aswath, M., Pandit, L. V., Kashyap, K., & Ramnath, R. (2016). Persistent genital arousal disorder. *Indian Journal of Psychological Medicine, 38*(4), 341–343. <https://doi.org/10.4103/0253-7176.185942>

[7] Battaglia, C., & Venturoli, S. (2009). Persistent genital arousal disorder and trazodone. Morphometric and vascular modifications of the clitoris. A case report. *The Journal of Sexual Medicine*, *6*(10), 2896–2900. <https://doi.org/10.1111/j.1743-6109.2009.01418.x>

[8] Bedell, S., Goldstein, A. T., & Burrows, L. (2014). A Periclitoral Mass as a Cause of Persistent Genital Arousal Disorder. *Journal of Sexual Medicine, 11*(1), 136–139. Scopus. <https://doi.org/10.1111/jsm.12165>

[9] Bell, C., Richardson, D., Goldmeier, D., Crowley, T., Kocsis, A., & Hill, S. (2007). Persistent sexual arousal in a woman with associated cardiac defects and raised atrial natriuretic peptide. *International Journal of STD & AIDS*, *18*(2), 130–131. <https://doi.org/10.1258/095646207779949592>

[10] Cohen, S. D. (2017). Diagnosis and Treatment of Persistent Genital Arousal Disorder. *Reviews in Urology, 19*(4), 265–267. <https://doi.org/10.3909/riu0784>

[11] Curran, K. A. (2019). Case Report: Persistent Genital Arousal Disorder in an Adolescent Woman. *Journal of Pediatric and Adolescent Gynecology*, *32*(2), 186–188. <https://doi.org/10.1016/j.jpag.2018.11.009>

[12] De Magalhaes, F. J. C., & Kumar, M. T. (2015). Persistent genital arousal disorder following selective serotonin reuptake inhibitor cessation. *Journal of Clinical Psychopharmacology, 35*(3), 352–354. <https://doi.org/10.1097/JCP.0000000000000318>

[13] Deka, K., Dua, N., Kakoty, M., & Ahmed, R. (2015). Persistent genital arousal disorder: Successful treatment with leuprolide (antiandrogen). *Indian Journal of Psychiatry, 57*(3), 326–328. <https://doi.org/10.4103/0019-5545.166633>

[14] Dikici, S., Gunal, D. I., Arslan, G., & Kayıkcı, M. A. (2015). Restless genital syndrome in a male patient relieved by pramipaxol and gabapentin. *Neurology Asia, 20*(4), 405–406.

[15] Eibye, S., & Jensen, H. M. (2014). Persistent genital arousal disorder: Confluent patient history of agitated depression, paroxetine cessation, and a tarlov cyst. *Case Reports in Psychiatry*, *2014*, 529052. <https://doi.org/10.1155/2014/529052>

[16] Elkins, G. R., Ramsey, D., & Yu, Y. (2014). Hypnotherapy for persistent genital arousal disorder: A case study. *The International Journal of Clinical and Experimental Hypnosis*, *62*(2), 215–223. <https://doi.org/10.1080/00207144.2014.869136>

[17] Gadit, A. (2013). Persistent genital arousal disorder: A clinical challenge. *BMJ Case Reports, 2013*, bcr2013009098. <https://doi.org/10.1136/bcr-2013-009098>

[18] Gündüz, N., Polat, A., & Turan, H. (2019). Persistent Genital Arousal Disorder Treated with Duloxetine: A Case Report. *Turkish journal of psychiatry, 30*(1), 67–70.

[19] Hiller, J., & Hekster, B. (2007). Couple therapy with cognitive behavioural techniques for persistent sexual arousal syndrome. *Sexual and Relationship Therapy*, *22*(1), 91–96. <https://doi.org/10.1080/14681990600815285>

[20] Hrynko, M., Kotas, R., Pokryszko-Dragan, A., Nowakowska-Kotas, M., & Podemski, R. (2017). Persistent genital arousal disorder—A case report. *Psychiatria Polska*, *51*(1), 117–124. <https://doi.org/10.12740/PP/64869>

[21] Jones, C. L., Fischer, J. R., & Hernandez, S. L. (2016). Sacral Neuromodulation for the Treatment of Persistent Genital Arousal Disorder. *Obstetrics and Gynecology, 128*(2), 321–323. <https://doi.org/10.1097/AOG.0000000000001452>

[22] Kamatchi, R., & Ashley-Smith, A. (2013). Persistent genital arousal disorder in a male: A case report and analysis of the cause. *British Journal of Medical Practitioners, 6*(1), a605.

[23] Korda, J. B., Pfaus, J. G., & Goldstein, I. (2009). Persistent genital arousal disorder: A case report in a woman with lifelong PGAD where serendipitous administration of varenicline tartrate resulted in symptomatic improvement. *The Journal of Sexual Medicine*, *6*(5), 1479–1486. <https://doi.org/10.1111/j.1743-6109.2008.01210.x>

[24] Korda, J. B., Pfaus, J. G., Kellner, C. H., & Goldstein, I. (2009). Persistent genital arousal disorder (PGAD): Case report of long-term symptomatic management with electroconvulsive therapy. *Journal of Sexual Medicine*, *6*(10), 2901–2909. <https://doi.org/10.1111/j.1743-6109.2009.01421.x>

[25] Krapf, J. M., & Goldstein, A. T. (2013). Two case presentations of profound labial edema as a presenting symptom of hypermobility-type ehlers-danlos syndrome. *Journal of Sexual Medicine, 10*(9), 2347–2350. <https://doi.org/10.1111/jsm.12229>

[26] Kruger, T. H. C., & Hartmann, U. (2016). A case of comorbid persistent genital arousal disorder and premature ejaculation: Killing two birds with one stone. *Journal of Sex & Marital Therapy, 42*(1), 1–3. <https://doi.org/10.1080/0092623X.2015.1053022>

[27] Leiblum, S., & Nathan, S. (2002). Persistent sexual arousal syndrome in women: A not uncommon but little recognized complaint. *Sexual and Relationship Therapy, 17*(2), 191–198. <https://doi.org/10.1080/14681990220121301>

[28] Mahoney, S., & Zarate Jr., C. (2007). Persistent sexual arousal syndrome: A case report and review of the literature. *Journal of Sex and Marital Therapy*, *33*(1), 65–71. <https://doi.org/10.1080/00926230600998532>

[29] Mariyam Farzana, S. F., Veera Goudhaman, T. S., & Pawan Kumar, M. S. (2018). Persistent genital arousal disorder intervention with manual physiotherapy-a case study. *Asian Journal of Pharmaceutical and Clinical Research, 11*(10), 3–4. <https://doi.org/10.22159/ajpcr.2018.v11i10.27327>

[30] McMullen, R., & Agarwal, S. (2016). Persistent genital arousal disorder-case report of symptomatic relief of symptoms with transcranial magnetic stimulation. *Journal of ECT, 32*(3), e9–e10. <https://doi.org/10.1097/YCT.0000000000000299>

[31] Miyake, K., Takaki, M., Sakamoto, S., Kawada, K., Inoue, S., & Yamada, N. (2018). Restless Genital Syndrome Induced by Milnacipran. *Clinical Neuropharmacology*, *41*(3), 109–110. <https://doi.org/10.1097/WNF.0000000000000279>

[32] Nazik, H., Api, M., Aytan, H., & Narin, R. (2014). A new medical treatment with botulinum toxin in persistent genital arousal disorder: Successful treatment of two cases. *Journal of Sex and Marital Therapy, 40*(3), 170–174. <https://doi.org/10.1080/0092623X.2013.788109>

[33] Philippsohn, S., & Kruger, T. H. C. (2012). Persistent genital arousal disorder: Successful treatment with duloxetine and pregabalin in two cases. *The Journal of Sexual Medicine, 9*(1), 213–217. <https://doi.org/10.1111/j.1743-6109.2011.02518.x>

[34] Ramic, M. (2013). A case of persistent genital arousal disorder successfully treated with topiramate in a physically healthy individual. *Journal of Clinical Psychiatry, 74*(7), 693. <https://doi.org/10.4088/JCP.12cr08280>

[35] Rosenbaum, T. Y. (2010). Physical therapy treatment of persistent genital arousal disorder during pregnancy: A case report. *Journal of Sexual Medicine*, *7*(3), 1306–1310. <https://doi.org/10.1111/j.1743-6109.2009.01654.x>

[36] Sforza, E., Hupin, D., & Roche, F. (2017). Restless genital syndrome: Differential diagnosis and treatment with pramipexole. *Journal of Clinical Sleep Medicine, 13*(9), 1109–1110. Scopus. <https://doi.org/10.5664/jcsm.6736>

[37] Stevenson, B. J., & Köhler, T. S. (2015). First reported case of isolated persistent genital arousal disorder in a male. *Case Reports in Urology, 2015*, 465748. <https://doi.org/10.1155/2015/465748>

[38] Waldinger, M. D., de Lint, G. J., Venema, P. L., van Gils, A. P., & Schweitzer, D. H. (2010). Successful transcutaneous electrical nerve stimulation in two women with restless genital syndrome: The role of aδ- and c-nerve fibers. *Journal of Sexual Medicine, 7*(3), 1190–1199. Scopus. <https://doi.org/10.1111/j.1743-6109.2009.01578.x>

[39] Waldinger, M. D., & Schweitzer, D. H. (2018). Restless genital syndrome (ReGS) should be distinguished from spontaneous orgasms: A case report of cannabis-induced spontaneous orgasm. *Journal of Sex & Marital Therapy*, *44*(3), 231–235. <https://doi.org/10.1080/0092623X.2017.1377130>

[40] Waldinger, M. D., Venema, P. L., van Gils, A. P., Schutter, E. M., & Schweitzer, D. H. (2010). Restless genital syndrome before and after clitoridectomy for spontaneous orgasms: A case Report. *Journal of Sexual Medicine*, *7*(2), 1029–1034. <https://doi.org/10.1111/j.1743-6109.2009.01571.x>

[41] Waldinger, M. D., Venema, P. L., Van Gils, A. P. G., De Lint, G. J., & Schweitzer, D. H. (2011). Stronger Evidence for Small Fiber Sensory Neuropathy in Restless Genital Syndrome: Two Case Reports in Males. *Journal of Sexual Medicine, 8*(1), 325–330. Scopus. <https://doi.org/10.1111/j.1743-6109.2010.02079.x>

[42] Wylie, K., Levin, R., Hallam-Jones, R., & Goddard, A. (2006). Sleep exacerbation of persistent sexual arousal syndrome in a postmenopausal woman. *Journal of Sexual Medicine, 3*(2), 296–302.. <https://doi.org/10.1111/j.1743-6109.2005.00167.x>

[43] Yero, S. A., McKinney, T., Petrides, G., Goldstein, I., & Kellner, C. H. (2006). Successful Use of Electroconvulsive Therapy in 2 Cases of Persistent Sexual Arousal Syndrome and Bipolar Disorder. *The Journal of ECT*, *22*, 274–275. <https://doi.org/10.1097/01.yct.0000244247.33038.26>

[44] Yildirim, E. A., Hacioglu Yıldırım, M., Kucukparlak, I., Bircan, I., Cicek, F., Essizoglu, A., … Erkiran, M. (2017). Case Reports of a Mother and Daughter Diagnosed With Persistent Genital Arousal Disorder. *Journal of Sex & Marital Therapy*, *43*(4), 295–297. <https://doi.org/10.1080/0092623X.2016.1232324>

[45] Zoorob, D., Deis, A. S., & Lindsay, K. (2019). Refractory Sexual Arousal Subsequent to Sacral Neuromodulation. *Case Reports in Obstetrics and Gynecology, 2019*, 7519164. https://doi.org/10.1155/2019/7519164

[46] Patricia, F. M., José, F. P., & Marcelo, M. (2015). Persistent Genital Arousal Disorder as a Dissociative Trauma Related Condition Treated with Brainspotting – A Successful Case Report. *International Journal of School and Cognitive Psychology*, *S1*, 1. <https://doi.org/10.4172/2469-9837.S1-002>

[47] Philippsohn, S. (2011). Persistent genital arousal disorder (PGAD): An overview of the disease and its treatment-Including two successfully treated cases. Sexuologie: *Zeitschrift für sexualmedizinische Fortbildung und Forschung, 18*, 48–56.

[48] Seifert, J., Bleich, S., Degner, D., Rüther, E., Renate, G., & Toto, S. (2020). „Persistent genital arousal disorder“ after discontinuation of sertraline. *Psychopharmakotherapie*, *27*(2), 77–80.

[49] Bilal, A. (2020). Treatment of persistent genital arousal disorder: Single case study. *Cogent Psychology*, *7*(1), 1849949. <https://doi.org/10.1080/23311908.2020.1849949>

[50] Ferenidou, F., Mourikis, I., Sotiropoulou, P., & Vaidakis, N. (2019). Zolpidem related persistent genital arousal disorder: An interesting case. *Psychiatriki, 30*(4), 339–344. <https://doi.org/10.22365/jpsych.2019.304.339>

[51] Dallagiacoma, S., Flora, G., Ferrone, S., Parodi, F., & Federico, A. (2020). Tarlov’s cyst as an underestimated cause of persistent genital arousal disorder: A case report and review. *Neurological Sciences, 41*(11), 3337–3339. <https://doi.org/10.1007/s10072-020-04504-4>

[52] Zwerling, B., Keymeulen, S., & Krychman, M. L. (2021). Sleep and Sex: A Review of the Interrelationship of Sleep and Sexuality Disorders in the Female Population, Through the Lens of Sleeping Beauty Syndrome. *Sexual Medicine Reviews, 9*(2), 221–229. <https://doi.org/10.1016/j.sxmr.2020.08.005>

[53] Feigenbaum, F., & Boone, K. (2015). Persistent Genital Arousal Disorder Caused by Spinal Meningeal Cysts in the Sacrum: Successful Neurosurgical Treatment. *Obstetrics and Gynecology*, *126*(4), 839–843. <https://doi.org/10.1097/AOG.0000000000001060>

[54] Gaines, N., Odom, B. D., Killinger, K. A., & Peters, K. M. (2018). Pudendal Neuromodulation as a Treatment for Persistent Genital Arousal Disorder-A Case Series. *Female Pelvic Medicine & Reconstructive Surgery*, *24*(4), e1–e5. <https://doi.org/10.1097/SPV.0000000000000435>

[55] Goldmeier, D., & Leiblum, S. (2008). Interaction of organic and psychological factors in persistent genital arousal disorder in women: A report of six cases. *International Journal of STD & AIDS*, *19*(7), 488–490. <https://doi.org/10.1258/ijsa.2007.007298>

[56] Klifto, K., & Dellon, A. L. (2020). Persistent genital arousal disorder: Treatment by neurolysis of dorsal branch of pudendal nerve. *Microsurgery*, *40*(2), 160–166. <https://doi.org/10.1002/micr.30464>

[57] Leiblum, S. R., & Chivers, M. L. (2007). Normal and persistent genital arousal in women: New perspectives. *Journal of Sex and Marital Therapy*, *33*(4), 357–373. <https://doi.org/10.1080/00926230701385605>

[58] Leiblum, S. R., & Goldmeier, D. (2008). Persistent genital arousal disorder in women: Case reports of association with anti-depressant usage and withdrawal. *Journal of Sex & Marital Therapy*, *34*(2), 150–159. <https://doi.org/10.1080/00926230701636205>

[59] Leiblum, S. R, & Nathan, S. G. (2001). Persistent sexual arousal syndrome: A newly discovered pattern of female sexuality. *Journal of Sex & Marital Therapy*, *27*(4), 365–380. <https://doi.org/10.1080/009262301317081115>

[60] Oaklander, A. L., Sharma, S., Kessler, K., & Price, B. H. (2020). Persistent genital arousal disorder: A special sense neuropathy. *Pain Reports*, *5*(1), e801. <https://doi.org/10.1097/PR9.0000000000000801>

[61] Yildirim, E. A., Hacioglu, M., Essizoglu, A., & Kucukparlak, I. (2012). Persistent genital arousal disorder misdiagnosed because of Islamic religious bathing rituals: A report of three cases. *Journal of Sex & Marital Therapy, 38*(5), 436–444. <https://doi.org/10.1080/0092623X.2011.606888>

[62] Yildirim, E. A., Hacioglu Yildirim, M., Carpar, E., & Sarac, I. (2017). Clomipramine trial for treatment-resistant persistent genital arousal disorder: A case series. *Journal of Psychosomatic Obstetrics and Gynaecology, 38(*4), 260–267. <https://doi.org/10.1080/0167482X.2017.1296427>

[63] Carvalho, J., Veríssimo, A., & Nobre, P. J. (2013). Cognitive and emotional determinants characterizing women with persistent genital arousal disorder. *The Journal of Sexual Medicine*, *10*(6), 1549–1558. <https://doi.org/10.1111/jsm.12122>

[64] Carvalho, J., Verissimo, A., & Nobre, P. J. (2015). Psychological factors predicting the distress to female persistent genital arousal symptoms. *Journal of Sex & Marital Therapy*, *41*(1), 11–24. <https://doi.org/10.1080/0092623X.2013.869776>

[65] Garvey, L. J., West, C., Latch, N., Leiblum, S., & Goldmeier, D. (2009). Report of spontaneous and persistent genital arousal in women attending a sexual health clinic. *International Journal of STD & AIDS*, *20*(8), 519–521. <https://doi.org/10.1258/ijsa.2008.008492>

[66] Healy, D., Le Noury, J., & Mangin, D. (2018). Enduring sexual dysfunction after treatment with antidepressants, 5α-reductase inhibitors and isotretinoin: 300 cases. *The International Journal of Risk & Safety in Medicine*, *29*(3–4), 125–134. <https://doi.org/10.3233/JRS-180744>

[67] Jackowich, R., Pink, L., Gordon, A., Poirier, É., & Pukall, C. F. (2018). Symptom Characteristics and Medical History of an Online Sample of Women Who Experience Symptoms of Persistent Genital Arousal. *Journal of Sex & Marital Therapy*, *44*(2), 111–126. <https://doi.org/10.1080/0092623X.2017.1321598>

[68] Jackowich, R. A., Pink, L., Gordon, A., Poirier, E., & Pukall, C. F. (2018). An online cross-sectional comparison of women with symptoms of persistent genital arousal, painful persistent genital arousal, and chronic vulvar pain. *Journal of Sexual Medicine, 15*(4), 558–567. <https://doi.org/10.1016/j.jsxm.2018.02.007>

[69] Jackowich, R. A., Poirier, E., & Pukall, C. F. (2020). A comparison of medical comorbidities, psychosocial, and sexual well-being in an online cross-sectional sample of women experiencing persistent genital arousal symptoms and a control group. *Journal of Sexual Medicine*, *17*(1), 69–82. <https://doi.org/10.1016/j.jsxm.2019.09.016>

[70] Komisaruk, B. R., & Lee, H.-J. (2012). Prevalence of Sacral spinal (Tarlov) cysts in persistent genital arousal disorder. *Journal of Sexual Medicine*, *9*(8), 2047–2056. <https://doi.org/10.1111/j.1743-6109.2012.02765.x>

[71] Leiblum, S., Brown, C., Wan, J., & Rawlinson, L. (2005). Persistent sexual arousal syndrome: A descriptive study. *Journal of Sexual Medicine, 2*(3), 331–337. <https://doi.org/10.1111/j.1743-6109.2005.20357.x>

[72] Leiblum, S., Seehuus, M., & Brown, C. (2007). Persistent genital arousal: Disordered or normative aspect of female sexual response? *The Journal of Sexual Medicine*, *4*(3), 680–689. <https://doi.org/10.1111/j.1743-6109.2007.00495.x>

[73] Leiblum, S., Seehuus, M., Goldmeier, D., & Brown, C. (2007). Psychological, medical, and pharmacological correlates of persistent genital arousal disorder. *The Journal of Sexual Medicine*, *4*(5), 1358–1366. <https://doi.org/10.1111/j.1743-6109.2007.00575.x>

[74] Leiblum, S. R., & Seehuus, M. (2009). FSFI scores of women with persistent genital arousal disorder compared with published scores of women with female sexual arousal disorder and healthy controls. *Journal of Sexual Medicine*, *6*(2), 469–473. <https://doi.org/10.1111/j.1743-6109.2008.01077.x>

[75] Pink, L., Rancourt, V., & Gordon, A. (2014). Persistent genital arousal in women with pelvic and genital pain. *Journal of Obstetrics and Gynaecology Canada*, *36*(4), 324–330. <https://doi.org/10.1016/S1701-2163(15)30608-3>

[76] Squibb, L., Stepleman, L., Goldstein, I., Sand, M., & Zamboni, B. (2019). Predictors and Moderators of Sexually Related Distress in Women with Persistent Genital Arousal Disorder. *International Journal of Sexual Health, 31*(4), 426–438. <https://doi.org/10.1080/19317611.2019.1674439>

[77] Villot, A., Thubert, T., Deffieux, X., Jousse, M., Breton, F. L., Lacroix, P., & Amarenco, G. (2016). Perineal Neurophysiological Assessment in 23 Patients Suffering from Persistent Genital Arousal Disorders: Evidence of Pudendal Neuropathy? *International Journal of Sexual Health*, *28*(1), 50–54. <https://doi.org/10.1080/19317611.2015.1080778>

[78] Waldinger, M. D, & Schweitzer, D. H. (2009). Persistent genital arousal disorder in 18 Dutch women: Part II-A syndrome clustered with restless legs and overactive bladder. *Journal of Sexual Medicine*, *6*(2), 482–497. <https://doi.org/10.1111/j.1743-6109.2008.01114.x>

[79] Waldinger, M. D, van Gils, A. P. G., Ottervanger, H. P., Vandenbroucke, W. V. A., & Tavy, D. L. J. (2009). Persistent genital arousal disorder in 18 Dutch women: Part I. MRI, EEG, and transvaginal ultrasonography investigations. *Journal of Sexual Medicine*, *6*(2), 474–481. <https://doi.org/10.1111/j.1743-6109.2008.01113.x>

[80] Waldinger, M. D., Venema, P. L., van Gils, A. P. G., & Schweitzer, D. H. (2009). New insights into restless genital syndrome: Static mechanical hyperesthesia and neuropathy of the nervus dorsalis clitoridis. *Journal of Sexual Medicine, 6*(10), 2778–2787. <https://doi.org/10.1111/j.1743-6109.2009.01435.x>

[81] Jackowich, R. A., & Pukall, C. F. (2020). Prevalence of Persistent Genital Arousal Disorder in 2 North American Samples. *The Journal of Sexual Medicine*, *17*(12), 2408–2416. <https://doi.org/10.1016/j.jsxm.2020.09.004>

[82] Dèttore, D., & Pagnini, G. (2021). Persistent Genital Arousal Disorder: A Study on an Italian Group of Female University Students. *Journal of Sex and Marital Therapy*, *47*(1), 60–79. <https://doi.org/10.1080/0092623X.2020.1804022>

[83] Bronner, G., Hassin-Baer, S., & Gurevich, T. (2017). Sexual Preoccupation Behavior in Parkinson’s Disease. *Journal of Parkinson’s Disease, 7*(1), 175–182. <https://doi.org/10.3233/JPD-160926>

[84] Brotto, L. A., Bitzer, J., Laan, E., Leiblum, S., & Luria, M. (2010). Women’s sexual desire and arousal disorders. *The Journal of Sexual Medicine, 7*(1 Pt 2), 586–614. <https://doi.org/10.1111/j.1743-6109.2009.01630.x>

[85] Dick, B., Natale, C., Reddy, A., Akula, K. P., Yousif, A., & Hellstrom, W. J. G. (2021). Application of Botulinum Neurotoxin in Female Sexual and Genitourinary Dysfunction: A Review of Current Practices. *Sexual Medicine Reviews, 9*(1), 57–63. <https://doi.org/10.1016/j.sxmr.2020.01.003>

[86] Facelle, T. M., Sadeghi-Nejad, H., & Goldmeier, D. (2013). Persistent genital arousal disorder: Characterization, etiology, and management. Journal of Sexual Medicine, 10(2), 439–450. <https://doi.org/10.1111/j.1743-6109.2012.02990.x>

[87] Giraldi, A., Rellini, A. H., Pfaus, J., & Laan, E. (2013). Female sexual arousal disorders. *Journal of Sexual Medicine, 10*(1), 58–73. <https://doi.org/10.1111/j.1743-6109.2012.02820.x>

[88] Goldmeier, D., & Leiblum, S. R. (2006). Persistent genital arousal in women—A new syndrome entity. *International Journal of STD and AIDS, 17*(4), 215–216. <https://doi.org/10.1258/095646206776253480>

[89] Goldmeier, D., Mears, A., Hiller, J., & Crowley, T. (2009). Persistent genital arousal disorder: A review of the literature and recommendations for management. *International Journal of STD & AIDS, 20*(6), 373–377. <https://doi.org/10.1258/ijsa.2009.009087>

[90] Higgins, A., Nash, M., & Lynch, A. M. (2010). Antidepressant-associated sexual dysfunction: Impact, effects, and treatment. *Drug, Healthcare and Patient Safety, 2*(1), 141–150. <https://doi.org/10.2147/DHPS.S7634>

[91] Jackowich, R. A., Pink, L., Gordon, A., & Pukall, C. F. (2016). Persistent Genital Arousal Disorder: A Review of Its Conceptualizations, Potential Origins, Impact, and Treatment. *Sexual Medicine Reviews*, *4*(4), 329–342. <https://doi.org/10.1016/j.sxmr.2016.06.003>

[92] Kingsberg, S. A., Althof, S., Simon, J. A., Bradford, A., Bitzer, J., Carvalho, J., … Shifren, J. L. (2017). Female sexual dysfunction-Medical and psychological treatments, Committee 14. *Journal of Sexual Medicine*, *14*, 1463–1491. <https://doi.org/10.1016/j.jsxm.2017.05.018>

[93] Klifto, K. M., & Dellon, A. L. (2020). Persistent Genital Arousal Disorder: Review of Pertinent Peripheral Nerves. *Sexual Medicine Reviews*, *8*(2), 265–273. <https://doi.org/10.1016/j.sxmr.2019.10.001>

[94] Kruger, T. H. C. (2018). Can pharmacotherapy help persistent genital arousal disorder? *Expert Opinion on Pharmacotherapy*, *19*(15), 1705–1709. <https://doi.org/10.1080/14656566.2018.1525359>

[95] Markos, A. R., & Dinsmore, W. (2013). Persistent genital arousal and restless genitalia: Sexual dysfunction or subtype of vulvodynia? *International Journal of STD and AIDS, 24*(11), 852–858. <https://doi.org/10.1177/0956462413489276>

[96] Parish, S. J., Goldstein, A. T., Goldstein, S. W., Goldstein, I., Pfaus, J., Clayton, A. H., … Whipple, B. (2016). Toward a more evidence-based nosology and nomenclature for female sexual dysfunctions-Part II. *Journal of Sexual Medicine*, *13*(12), 1888–1906. <https://doi.org/10.1016/j.jsxm.2016.09.020>

[97] Pernot-Masson, A. C. (2020). Persistent genital arousal disorder: A neurodevelopmental hypothesis. *European Journal of Trauma & Dissociation*, *4*(3), 100159. <https://doi.org/10.1016/j.ejtd.2020.100159>

[98] Pukall, C. F., Jackowich, R., Mooney, K., & Chamberlain, S. M. (2019). Genital Sensations in Persistent Genital Arousal Disorder: A Case for an Overarching Nosology of Genitopelvic Dysesthesias? *Sexual Medicine Reviews*, *7*(1), 2–12. <https://doi.org/10.1016/j.sxmr.2018.08.001>

[99] Schenck, C. H., Amulf, I., & Mahowald, M. W. (2007). Sleep and sex: What can go wrong? A review of the literature on sleep related disorders and abnormal sexual behaviors and experiences. *Sleep, 30*(6), 683–702. Scopus. <https://doi.org/10.1093/sleep/30.6.683>

[100] Towe, M., La, J., El-Khatib, F., Roberts, N., Yafi, F. A., & Rubin, R. (2020). Diet and Female Sexual Health. *Sexual Medicine Reviews, 8*(2), 256–264. <https://doi.org/10.1016/j.sxmr.2019.08.004>

[101] Yafi, F. A., April, D., Powers, M. K., Sangkum, P., & Hellstrom, W. J. G. (2015). Penile Priapism, Clitoral Priapism, and Persistent Genital Arousal Disorder: A Contemporary Review. *Sexual Medicine Reviews, 3*(3), 145–159. <https://doi.org/10.1002/smrj.51>

[102] Basson, R., Leiblum, S., Brotto, L., Derogatis, L., Fourcroy, J., Fugl‐Meyer, K., … Schultz, W. W. (2004). Revised Definitions of Women’s Sexual Dysfunction. *The Journal of Sexual Medicine, 1*(1), 40–48. <https://doi.org/10.1111/j.1743-6109.2004.10107.x>

[103] Jackowich, R. A., & Pukall, C. F. (2020). Persistent Genital Arousal Disorder: A Biopsychosocial Framework. *Current Sexual Health Reports*, *12*, 127–135. <https://doi.org/10.1007/s11930-020-00268-2>
